# Long-term risk of tuberculosis among individuals with Xpert Ultra trace screening results in Uganda: a longitudinal follow-up study

**DOI:** 10.1016/S1473-3099(25)00536-5

**Published:** 2026-02

**Authors:** Joowhan Sung, Mariam Nantale, Annet Nalutaaya, Patrick Biché, James Mukiibi, Joab Akampurira, Rogers Kiyonga, Francis Kayondo, Michael Mukiibi, Caitlin Visek, Caleb E Kamoga, David W Dowdy, Achilles Katamba, Emily A Kendall

**Affiliations:** a Division of Infectious Diseases, Johns Hopkins University School of Medicine, Baltimore, MD, USA; b Uganda Tuberculosis Implementation Research Consortium, Walimu, Kampala, Uganda; c Johns Hopkins Bloomberg School of Public Health, Department of Epidemiology, Baltimore, MD, USA; d Makerere University College of Health Science, Department of Internal Medicine, Clinical Epidemiology and Biostatistics Unit, Kampala, Uganda

## Abstract

**Background:**

Systematic screening for tuberculosis using Xpert Ultra can generate trace results of uncertain significance. Additional microbiological testing in this context is often negative, but untreated individuals might still progress to culture-positive disease. We aimed to estimate the 2-year risk of tuberculosis among screened participants with trace-positive sputum (PWTS).

**Methods:**

In this longitudinal follow-up study, we conducted Ultra-based systematic screening for tuberculosis in Kampala, Uganda, from Feb 2, 2021, to April 27, 2024, enrolling PWTS as well as participants who were Ultra-positive or Ultra-negative controls. Recruitment occurred primarily through community-based screening events and door-to-door screening. Ultra sputum testing was offered to individuals aged 15 years or older who were not on active tuberculosis treatment, regardless of their symptoms. All PWTS, as well as age-matched and sex-matched participants with negative screening results and consecutive participants with positive screening results, were recruited. Participants underwent extensive initial evaluation, and untreated PWTS and negative-control participants were followed up with re-testing for up to 24 months. Our primary outcome was the cumulative hazard of tuberculosis among PWTS, using two definitions of tuberculosis: one incorporating clinician judgement and one strictly microbiological. We then compared hazards between PWTS and negative-control participants. We also assessed whether the presence of symptoms or chest x-ray abnormalities at baseline were associated with tuberculosis diagnosis during follow-up in PWTS.

**Findings:**

We screened 31 505 people for tuberculosis in Uganda using sputum Xpert Ultra as an initial test through event-based and door-to-door screening. We enrolled 128 PWTS and 139 age-matched and sex-matched control participants who were Ultra-negative (negative-control participants) into prospective cohorts and 110 control participants who were Ultra-positive (more than trace) for cross-sectional comparison. Of 128 PWTS, 79 (62%) were male, 49 (38%) were female, and 19 (15%) were HIV positive; 45 (35%) were recommended for treatment upon enrolment, eight (6%) were lost to follow-up within 3 months, and 75 (56%) were followed up for a median of 706 days (IQR 344–714), of whom 19 (25%) were recommended for treatment during follow-up. The cumulative hazard of tuberculosis among PWTS not treated at baseline was 0·24 (95% CI 0·15–0·40) at 1 year and 0·33 (0·21–0·54) at 2 years, versus 0·03 (0·01–0·10) at 2 years for negative-control participants. Hazards were similar for microbiologically defined tuberculosis (0·36 [95% CI 0·22–0·58] for PWTS *vs* 0·02 [0·01–0·10] for negative-control participants at 2 years). Tuberculosis diagnosis during follow-up was strongly associated with atypical baseline chest x-ray (ie, interpreted by radiologists as having any abnormality; hazard ratio 14·6 [95% CI 3·3–63·8]) but not with baseline symptoms (cough, fever, night sweats, or weight loss).

**Interpretation:**

Individuals with trace-positive sputum during screening have a substantial 2-year risk of tuberculosis, even when extensive initial evaluations do not confirm disease. Treatment should be considered for most screening participants with trace-positive sputum and atypical chest imaging.

**Funding:**

National Institutes of Health and the Gates Foundation.

## Introduction

Despite being a curable disease, tuberculosis continues to be the leading single-agent infectious cause of death worldwide, accounting for 1·25 million deaths in 2023.^1^ About 25% of individuals who develop tuberculosis are never diagnosed and started on treatment.^1^ Detecting tuberculosis at early stages through effective diagnostic strategies could reduce transmission and prevent severe outcomes.

Tuberculosis programmes are increasingly conducting community-based tuberculosis screening,^2^ and during such screening efforts, Xpert MTB/RIF Ultra (Cepheid, Sunnyvale, CA, USA), a molecular diagnostic tool for tuberculosis, is widely used as a confirmatory test for individuals with tuberculosis symptoms or atypical chest x-rays. In screening contexts, a large proportion of positive Ultra results are in the trace category.^3^, ^4^, ^5^ Trace results, which represent the lowest level of *Mycobacterium tuberculosis* DNA detection, are reported when the Ultra assay detects one of its multicopy amplification targets (IS1660 or IS1810) but not its single-copy *rpoB* gene target.^6^ Studies that have used Ultra as an initial or confirmatory test during general population screening in high-burden settings have reported that 24–46% of positive results are trace-positive.^3^, ^4^, ^5^, ^7^

**Research in context**

**Evidence before this study**

Within the past 5 years, advances in tuberculosis research have shifted the disease framework from a binary classification of latent versus active tuberculosis to a continuum of disease states. They have also led to a better understanding of the dynamic disease course of early tuberculosis, which can either progress to culture-positive disease or regress spontaneously over time. Trace results from Xpert MTB/RIF Ultra are sometimes perceived as false positives in individuals who subsequently test negative on additional diagnostic assays. However, some of these individuals could have early tuberculosis that falls below the detection threshold of existing diagnostic tests and could progress to microbiologically detectable disease over time. In screening contexts, trace-positive Ultra results constitute up to half of all positive results, and understanding how to interpret this result from screening participants, particularly among individuals without further evidence of tuberculosis on additional testing, is crucial. To investigate Ultra trace results, we searched PubMed for studies published in English from database inception up to July 10, 2025, using the terms “tuberculosis” AND (“Xpert” OR “Xpert Ultra” OR “Ultra”) AND “Trace” and also reviewed the reference lists of relevant search results. In two prevalence surveys that each used a combination of symptoms and chest x-rays as the screening test with Ultra as a confirmatory test, the proportion with positive cultures among those with trace-positive sputum ranged from 20% to 38%. In a study conducted in Uganda in which Ultra was used as an initial screening test, only 14% of individuals with a trace-positive result had positive sputum cultures. However, previous studies were limited to a one-time evaluation of individuals following a trace result. No one longitudinally followed up these individuals after a negative initial investigation to assess their long-term risk of tuberculosis and its predictors. The appropriate management strategy for such individuals remains unknown.

**Added value of this study**

To our knowledge, this is the first study that longitudinally followed up individuals with Ultra trace-positive screening results and estimated their risk of tuberculosis disease over time. In this study, individuals with Ultra trace-positive screening results who were not started on treatment after extensive diagnostic testing were followed up for up to 2 years with repeated testing. The 2-year cumulative hazard of tuberculosis disease was substantial at 0·33 (95% CI 0·21–0·54), compared with 0·03 (0·01–0·10) among individuals with Ultra-negative screening results. Those who had a typical chest x-ray at enrolment were at significantly lower risk of developing tuberculosis compared with those who had an atypical chest x-ray. 2-year tuberculosis risk was similar between those who reported symptoms at the time of enrolment and those who did not.

**Implications of all the available evidence**

The elevated 2-year risk of tuberculosis observed among people with trace results in this study, even when Ultra was used without any previous screening step, supports the provision of treatment for tuberculosis disease to most individuals who receive trace results during tuberculosis screening interventions, particularly when trace results were preceded by atypical chest x-ray. These results also show that x-ray could be a useful tool to guide treatment decision making for individuals with trace-positive sputum.

Due to lower tuberculosis prevalence among general populations compared with patients who are symptomatic and tested at health facilities, the positive predictive value of a trace result could be lower in screening contexts, but the proportion of trace results that reflect underlying tuberculosis disease is uncertain. We previously reported on systematic screening for tuberculosis that we conducted in Uganda using Ultra as an initial test regardless of symptoms, with detailed baseline evaluation of participants who received trace results.^5^ Among the first 92 participants, only 22 (24%) had microbiologically confirmed tuberculosis, with just 13 (14%) having a positive culture. However, tuberculosis has a dynamic and varied disease course, with progression and regression of bacterial burden occurring across different states of infection.^8^, ^9^, ^10^ We hypothesised that some individuals with trace-positive sputum who are otherwise microbiologically negative could have early (or otherwise minimal-burden) disease that could progress over time, such that they might benefit from preventive or empirical treatment. No existing studies have evaluated the long-term risk of tuberculosis among individuals with trace-positive sputum who otherwise do not have evidence of tuberculosis. Therefore, within the same cohort after additional enrolment, we aimed to estimate the 2-year risk of tuberculosis disease among participants with trace-positive screening results who had completed detailed evaluations without definite evidence of tuberculosis.

## Methods

### Study design and participants

In this longitudinal follow-up study, we conducted Ultra-based systematic screening for tuberculosis in Kampala, Uganda, from Feb 2, 2021, to April 27, 2024, enrolling participants with trace-positive sputum (PWTS) as well as control participants who were Ultra-positive or Ultra-negative. Procedures for recruitment, described previously,^5^ were continued until April 27, 2024, and are detailed in the appendix (p 3). Briefly, recruitment occurred primarily through community-based screening events and door-to-door screening and, to a lesser extent, through contact investigations. Ultra sputum testing was offered to individuals aged 15 years or older who were not on active tuberculosis treatment, regardless of their symptoms. Upon receiving the Ultra results from the study's screening activities, we recruited all PWTS, as well as age-matched and sex-matched participants with negative screening results (negative-control participants) and consecutive participants with positive (more than trace) screening results (positive-control participants) for further diagnostic evaluation (appendix p 4). To enhance our sample size, we also enrolled people who had trace-positive sputum during community-based, symptom-agnostic Ultra testing that was offered in high-risk areas of Kampala during national screening campaigns in September, 2022, March, 2023, September, 2023, and March, 2024.^11^ Tuberculosis screening continued until 128 PWTS were enrolled. Written informed consent (or adolescent assent with parental consent) was obtained from all study participants. The institutional review boards of the Johns Hopkins University School of Medicine (IRB00269370) and the Makerere University School of Public Health (Protocol 901) approved the study. The protocol is available in the appendix (pp 28–56).

### Procedures

**Figure 1:**
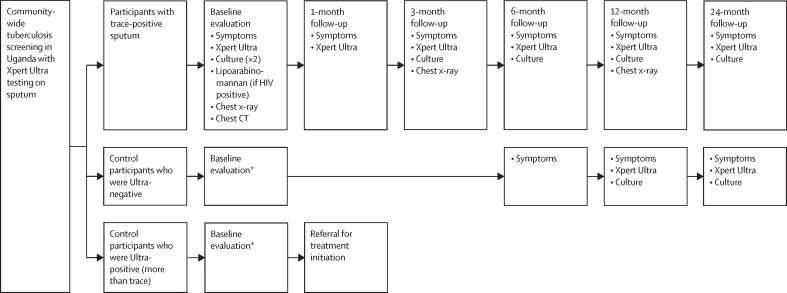
Study procedures for evaluation and follow-up after a trace Ultra result during community-wide screening *Negative-control and positive-control participants underwent the same baseline evaluation as participants with trace-positive sputum.

Upon enrolment, PWTS completed a standardised survey (including self-report of age, sex [male or female], race, and symptoms [appendix pp 7–8]), as well as extensive diagnostic testing by the study team, including repeat sputum Ultra, two sets of sputum liquid and solid mycobacterial cultures, HIV testing (using a serial rapid testing algorithm^12^ if not known positive, with linkage to care if positive), chest x-ray, and chest CT. Participants with HIV also received urine lipoarabinomannan testing (Determine TB LAM; Abbott, Abbott Park, IL, USA). Participants screened after June 7, 2021, were also surveyed about tuberculosis symptoms at the time of screening (appendix p 9). A panel of experienced Ugandan pulmonologists and radiologists (ie, consultants) regularly reviewed new results—including clinical risk factors, symptoms, laboratory results, and chest imaging—for PWTS and for negative-control participants with atypical findings, and made recommendations for additional diagnostic testing and initiation of treatment. PWTS who were not recommended for tuberculosis treatment or declined treatment had scheduled follow-up re-evaluations at months 1, 3, 6, 12, and 24 (figure 1), in addition to unscheduled re-evaluations as needed. At each scheduled follow-up visit, participants were assessed for symptoms and asked to provide expectorated sputum for repeat testing by Ultra. Chest x-rays were repeated at months 3 and 12, and sputum cultures were repeated for all PWTS at months 3 and 12, and additionally at months 6 and 24 after a November, 2023, protocol revision. Both negative-control and positive-control participants underwent the same diagnostic evaluations at baseline. Negative-control participants not recommended for treatment were followed up for up to 2 years, with symptom assessment at 6 months and repeat sputum testing at months 12 and 24; one negative-control participant with a trace result on repeat Ultra testing received more frequent follow-up similar to PWTS. Positive-control participants were referred for treatment initiation.

Chest x-ray and CT images of study participants were reviewed by two independent radiologists, with a third radiologist reviewing images with discrepant readings. The radiologists, masked to clinical information, assessed whether specified abnormalities (eg, nodule, cavity, and fibrosis) were present, and rated the consistency with current tuberculosis and (separately) with previous tuberculosis on four-point scales. These scores were averaged across all radiologists for reporting (appendix p 10). Chest x-ray images with any abnormalities were considered atypical. In addition, chest x-rays were retrospectively analysed with computer-aided detection (CAD) software (qXR version 4, Qure.ai, Mumbai, India); CAD results were unavailable during treatment decision making.

Our primary analysis defined tuberculosis based on treatment recommendation. Under this definition (chosen as the primary analysis for the breadth of evidence it considered; appendix p 11), all participants for whom consultants recommended treatment or an external clinician prescribed treatment were counted as having tuberculosis, including those diagnosed clinically. Participants with positive microbiological results that clinicians or consultants interpreted as false-positive were considered not to have tuberculosis under this definition. In secondary analyses, tuberculosis was defined microbiologically—specifically as having a positive sputum Ultra (required to be higher than trace to diagnose based on Ultra alone), a positive sputum culture for *M tuberculosis* complex, or a positive urine lipoarabinomannan among people with HIV. For survival analyses using microbiological positivity to define tuberculosis outcomes, we censored participants who initiated treatment without microbiological confirmation.

### Outcomes

The primary outcome was the cumulative cause-specific hazards of tuberculosis diagnosis during follow-up among PWTS who were not diagnosed at baseline using two definitions of tuberculosis (based on treatment recommendation and microbiologically). The prespecified secondary outcomes were hazard ratios (HRs) of tuberculosis diagnosis during follow-up comparing PWTS to negative-control participants and between subgroups of PWTS with different baseline characteristics.

### Statistical analysis

The sample size was chosen to have 80% power to identify an elevated tuberculosis diagnosis risk of approximately 4% per year among PWTS compared with negative-control participants (appendix pp 5–6). We first estimated the cumulative proportion of all PWTS who were recommended for treatment over the course of the study, with a corresponding 95% Wilson score CI. Participants lost to follow-up before 3 months were excluded from this analysis. Then, we used survival analysis to evaluate the risk of tuberculosis diagnosis during follow-up among those not diagnosed at baseline, and compared PWTS to negative-control participants. Participants were classified as having tuberculosis at baseline if their diagnosis was based on the enrolment evaluation, including positive baseline cultures that resulted weeks later. We estimated the cumulative cause-specific hazards of tuberculosis diagnosis among PWTS and among negative-control participants using the Nelson-Aalen estimator^13^ (appendix p 12). We then estimated HRs and corresponding 95% CIs to compare the two groups using a Cox proportional hazards model. We performed this analysis for both definitions of tuberculosis, using the corresponding definition of tuberculosis to also define the cohort without tuberculosis at baseline. A sensitivity analysis repeated the same analyses using only matched pairs (appendix p 4).

Using similar methods, we estimated the cumulative hazards of tuberculosis diagnosis during follow-up among subgroups of PWTS with different baseline characteristics, and calculated HRs for baseline risk factors, including male sex, HIV infection, positive symptom status (based on a four-symptom assessment: any cough, fever, night sweats, or weight loss), and history of previous tuberculosis treatment. We also estimated HRs comparing those with atypical versus typical baseline chest imaging by each of several definitions, namely (1) chest x-ray read by radiologists as having any abnormalities (ie, atypical chest x-ray), (2) chest x-ray read as suggestive of tuberculosis, (3) chest x-ray with qXR tuberculosis score (CAD score) of 0·2 or more, (4) chest x-ray with CAD score of 0·5 or more, and (5) CT read as suggestive of tuberculosis. All comparisons between subsets of PWTS were performed for each of two definitions of tuberculosis in parallel, and those with missing data for a given risk factor were excluded.

Finally, we compared baseline demographic, clinical, and radiographical characteristics among the following five groups of participants: (1) positive-control participants, (2) PWTS recommended for treatment at baseline (trace, tuberculosis at baseline), (3) PWTS recommended for treatment during follow-up (trace, tuberculosis during follow-up), (4) PWTS not recommended for treatment and followed up for at least 3 months (trace, no tuberculosis), and (5) negative-control participants. The CAD scores of baseline chest x-rays were compared between the five groups using Kruskall-Wallis tests followed by Dunn tests with Bonferroni correction.^14^ We estimated the area under the curve (AUC) of the baseline CAD score for any tuberculosis (diagnosed at baseline or during follow-up, using two definitions), among PWTS who were either diagnosed or followed up for at least 3 months. In the AUC analysis using the microbiological definition, participants with microbiologically unconfirmed tuberculosis were considered tuberculosis negative. This analysis was conducted after all participants had sufficient time to complete 12 months of follow-up (appendix p 13). Statistical significance was determined as a two-sided α less than 0·05. Analyses were performed using Stata version 16.1 and R version 4.3.2.

### Role of the funding source

The funders of the study had no role in the study design, data collection, data analysis, data interpretation, writing of the report, or the decision to submit.

## Results

**Figure 2:**
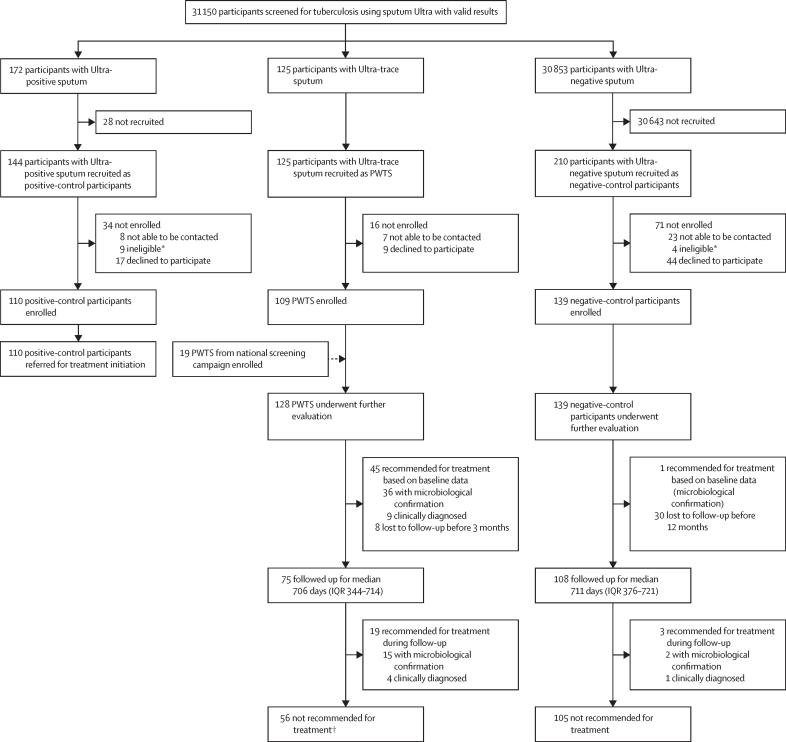
Recruitment and diagnostic outcomes PWTS=participants with trace-positive sputum. *Reasons for ineligibility are listed in the appendix (p 27). †Among PWTS, there were two participants at baseline and four during follow-up with presumed false-positive microbiological results, for whom treatment was not recommended.

A total of 31 505 individuals aged 15 years or older consented to tuberculosis screening. 16 568 individuals were recruited through event-based screening, 14 782 through door-to-door screening, and 155 through contact investigation. Of these, 31 321 (99·4%) successfully provided sputum samples, and 31 150 (98·9%) had valid Ultra results. Among 31 150 valid results, 297 (1·0%) were positive, and 125 (42%) of 297 results were trace positive. We recruited 109 (87%) of 125 PWTS and additionally recruited 19 participants who had trace-positive sputum during a national screening campaign,^11^ resulting in 128 PWTS enrolled (figure 2). We also enrolled 139 matched control participants who were Ultra-negative and 110 (76%) of 144 eligible people who were positive.

Among 128 PWTS, the median age was 33 (IQR 24–39), 79 (62%) were male, 49 (38%) were female, all were of Black African ethnicity, 19 (15%) had HIV, and 42 (46%) of 91 PWTS who were assessed reported any cough at screening (table 1). Of 128 PWTS, 45 (35%) were recommended tuberculosis treatment based on additional testing performed at baseline, including 36 with microbiological confirmation and 27 with positive culture (appendix pp 14–15). Of 45 PWTS recommended treatment at baseline, 34 (77%) had atypical chest x-ray, 41 (91%) had atypical chest CT, and one (2%) did not complete imaging studies. Eight (6%) PWTS who were not recommended treatment were lost to follow-up before 3 months.

**Table 1:** Characteristics of individuals screened for tuberculosis

		**Positive Ultra (n=110)**	Trace Ultra (n=120)*			**Negative Ultra (n=139)**
			Treatment recommended at baseline (n=45)	Treatment recommended during follow-up (n=19)	Treatment not recommended (n=56)*	
Age (years)†		35 (28–42)	35 (24–42)	32 (27–39)	33·5 (25–38)	32 (25–39)
Sex†						
	Female	26 (24%)	13 (29%)	7 (37%)	25 (45%)	60 (43%)
	Male	84 (76%)	32 (71%)	12 (63%)	31 (55%)	79 (57%)
HIV positive†		23 (21%)	9 (20%)	4 (21%)	6 (11%)	11 (8%)
HIV positive, not on antiretroviral therapy at enrolment		4 (4%)	1 (2%)	2 (11%)	0	1 (1%)
Household tuberculosis exposure within 1 year		15 (14%)	7 (16%)	4 (21%)	7 (13%)	7 (5%)
Screened during contact investigation		5 (5%)	2 (4%)	0	3 (5%)	0
Previous tuberculosis		20 (18%)	4 (9%)	7 (37%)	12 (21%)	6 (4%)
Previous tuberculosis within 2 years		13 (12%)	3 (7%)	3 (16%)	5 (9%)	3 (2%)
Currently smoking		36 (33%)	14 (31%)	7 (37%)	8 (14%)	16 (12%)
Underweight (BMI <18·5 kg/m^2^)		27/102 (26%)	17/45 (38%)	2/18 (11%)	4/46 (9%)	11/125 (9%)
Cough at screening‡		67/94 (71%)	21/35 (60%)	3/9 (33%)	18/41 (44%)	35/120 (29%)
Cough at enrolment		98 (89%)	37 (82%)	13 (68%)	37 (66%)	30 (22%)
	Weeks of cough§	6 (3–16)	4 (2–8)	2 (1–6)	3 (1–12)	2 (1–4)
	Cough severity on 0–100 visual analog scale§^15^	50 (30–70)	40 (20–60)	30 (10–50)	30 (20–50)	28 (10–30)
Any tuberculosis symptom at screening¶‡		68/94 (72%)	22/35 (63%)	4/9 (44%)	20/41 (49%)	40/120 (33%)
Any tuberculosis symptom at enrolmentll		104 (95%)	40 (89%)	17 (89%)	47 (84%)	58 (42%)
Positive QuantiFERON		59/67 (88%)	35/40 (88%)	12/15 (80%)	32/53 (60%)	51/106 (48%)
C-reactive protein ≥5·0 mg/L		56/103 (54%)	19/44 (43%)	4/18 (22%)	13/56 (23%)	21/136 (15%)

Data are median (IQR), n (%), or n/N (%). All participants were of Black African ethnicity.

* Excludes eight participants who completed less than 3 months of follow-up.

† Matching constrained the age and sex of negative controls to be similar to, and HIV prevalence to be no greater than, that of participants with trace-positive sputum as a whole.

‡ At the time of initial screening, before receiving Ultra result; asked of all individuals screening after June, 2021.

§ If cough reported.

¶ Any cough, fever, night sweats, or weight loss at the time of screening. llAny cough, fever, night sweats, or weight loss (5 kg over 12 months) at the time of enrolment.

The cohort analysed for tuberculosis diagnosis during follow-up consisted of 75 PWTS, with no loss to follow-up before 3 months. They were followed up for a median of 706 days (IQR 344–714), during which time 19 (25%) of 75 PWTS were recommended for tuberculosis treatment during follow-up, including 15 with microbiological confirmation (and four with microbiologically unconfirmed diagnoses, who were thus censored from the secondary analysis using a microbiologically defined outcome; appendix pp 16–17). Therefore, when we considered diagnosis cumulatively from enrolment among all 120 PWTS with sufficient follow-up data, 64 (53% [95% CI 44–62]) were recommended for tuberculosis treatment either at baseline or during follow-up. Three deaths occurred among study participants: one PWTS, one negative-control participant, and one positive-control participant (appendix p 13).

**Figure 3:**
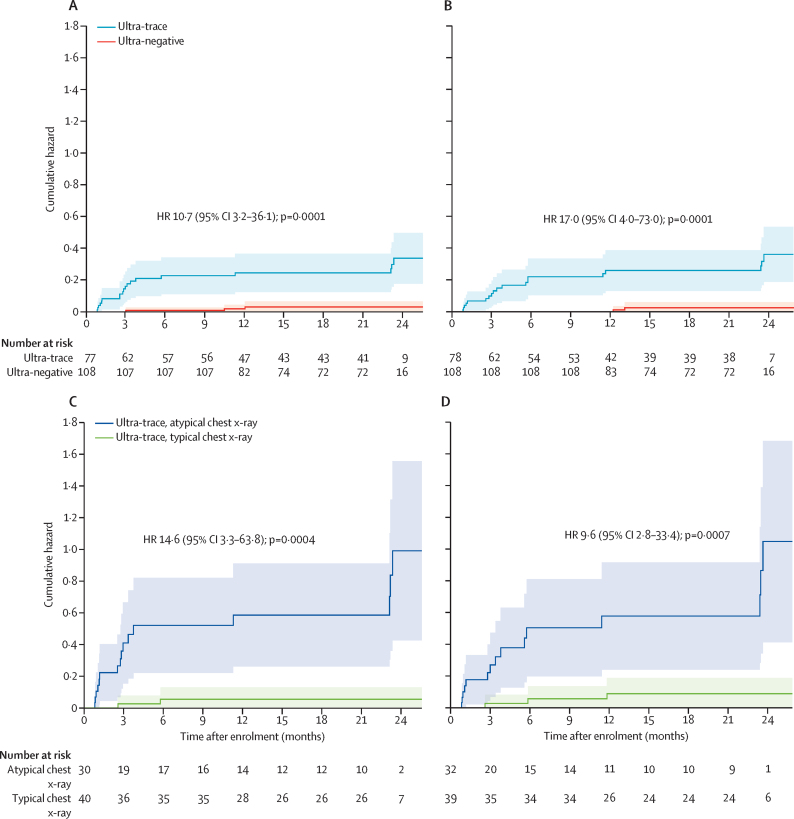
Cumulative cause-specific hazards of receiving a tuberculosis treatment recommendation or developing a positive microbiological result for tuberculosis Tuberculosis treatment recommendation (A) and positive microbiological result for tuberculosis (B) during follow-up in participants not diagnosed at baseline, estimated with the negative log transformation of Kaplan–Meier survival curves and stratified by participants' initial Ultra results during community-wide tuberculosis screening. Tuberculosis treatment recommendation (C) and positive microbiological result for tuberculosis (D) only for participants with initial trace-positive Ultra results, stratified by chest x-ray results at enrolment, as interpreted by human readers. Shaded areas represent 95% CI. HR=hazard ratio.

Among PWTS not diagnosed with tuberculosis through baseline evaluation, the cumulative cause-specific hazard of tuberculosis diagnosis during follow-up was 0·24 (95% CI 0·15–0·40) at 1 year and 0·33 (0·21–0·54) at 2 years when estimated based on treatment recommendation. Corresponding cumulative hazards based on microbiological positivity were 0·26 (0·16–0·42) at 1 year and 0·36 (0·22–0·58) at 2 years. The 2-year cumulative hazard among negative-control participants was 0·03 (0·01–0·10) based on treatment recommendation or 0·02 (0·01–0·10) based on microbiological positivity (figure 3A, B). Results restricted to matched pairs were similar (appendix p 18). Among PWTS not diagnosed at baseline, tuberculosis diagnosis during follow-up was strongly associated with atypical baseline chest x-ray as interpreted by radiologists (HR 14·6 [95% CI 3·3–63·8]; p=0·0004; figure 3C, D) but not with baseline cough (HR 1·2 [0·4–3·1]; p=0·73; appendix p 19). For sex, HIV status, presence of any tuberculosis symptom, and history of previous tuberculosis, HRs for tuberculosis diagnosis during follow-up were elevated (>1) but did not reach statistical significance in the primary analysis (appendix p 22).

**Figure 4:**
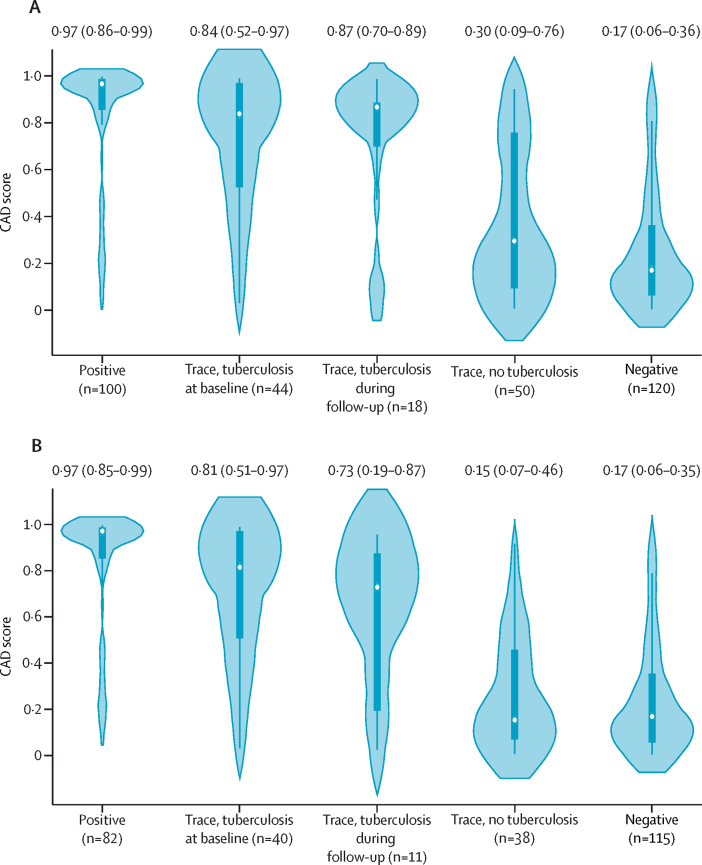
Distribution of CAD-interpreted baseline chest x-ray scores by initial sputum Ultra results and tuberculosis treatment recommendation Categorised into five groups: positive-control participants, PWTS and tuberculosis diagnosed at baseline, PWTS and tuberculosis diagnosed during follow-up, PWTS not diagnosed with tuberculosis, and negative-control participants. Curves represent the distribution of CAD scores based on kernel density estimates. Dots indicate median values, boxes show IQR, and whiskers extend to the upper and lower adjacent values for CAD scores in each group. The medians and IQRs of CAD scores for each group are listed above each plot. (A) All study participants. (B) Participants without a history of tuberculosis. CAD=computer-aided detection. PWTS=participants with trace-positive sputum.

The median CAD scores (by qXR) were highest among positive-control participants (0·97 [IQR 0·86–0·99]) and lowest among negative-control participants (0·17 [0·06–0·36]). PWTS diagnosed at baseline and during follow-up had similar median CAD scores (0·84 [0·52–0·97] *vs* 0·87 [0·70–0·89]); PWTS followed up for at least 3 months without tuberculosis diagnosis had lower CAD scores (0·30 [0·09–0·76]) than PWTS with tuberculosis diagnosis at baseline (p=0·0001) or during follow-up (p=0·055). Among PWTS not diagnosed with tuberculosis, elevated CAD scores occurred mainly in those with history of previous tuberculosis treatment (figure 4; appendix p 23).

Baseline chest x-ray interpretation by CAD had an AUC of 0·77 (95% CI 0·69–0·86) for predicting tuberculosis at baseline or during follow-up among PWTS. AUC did not change when tuberculosis was defined microbiologically (AUC 0·77 [95% CI 0·68–0·86]; appendix p 24). A baseline CAD score of 0·5 had a sensitivity of 77% (95% CI 66–86) for tuberculosis within 2 years (including baseline diagnoses) using a definition based on treatment recommendation and 75% (62–84) using a microbiological definition, with 64% (50–76) specificity using either definition (appendix p 25). The AUC estimates were numerically higher in PWTS without a previous history of tuberculosis treatment compared with those with a history of tuberculosis (AUC 0·84 [95% CI 0·76–0·92] *vs* 0·70 [0·47–0·92] for treatment recommendation [p=0·24]; 0·80 [0·70–0·89] *vs* 0·75 [0·53–0·97] for microbiological positivity [p=0·69]).

Of 62 PWTS recommended treatment at baseline or during follow-up who completed a chest x-ray at baseline, only four (6%) had very low qXR scores (<0·1); all four participants had their baseline x-rays interpreted as typical by radiologists. One of them had a typical baseline chest CT but had untreated HIV and was started on tuberculosis treatment by her HIV clinician. The remaining three participants, all of whom had culture-confirmed tuberculosis, had subcentimetre nodules on their baseline chest CTs. Nearly all PWTS diagnosed with tuberculosis at baseline or during follow-up (60 [95%] of 63 PWTS diagnosed with tuberculosis) had atypical chest CTs at baseline (table 2), compared with 19 (36%) of 53 PWTS who were not diagnosed.

**Table 2:** Baseline chest CT characteristics of individuals screened for tuberculosis who completed chest CT at baseline (n=325)

		**Positive Ultra (n=101)**	**Trace Ultra (n=116)**			**Negative Ultra (n=108)**
			Diagnosed at baseline (n=44)	Diagnosed during follow-up (n=19)	Not diagnosed (n=53)	
Any abnormality		96 (95%)	41 (93%)	19 (100%)	19 (36%)	24 (22%)
	Consolidation	55 (54%)	20 (45%)	4 (21%)	4 (8%)	3 (3%)
	Ground glass opacity	32 (32%)	11 (25%)	3 (16%)	3 (6%)	4 (4%)
	Nodule (≤3 cm)	89 (88%)	34 (77%)	19 (100%)	14 (26%)	9 (8%)
	Mass (>3 cm)	3 (3%)	1 (2%)	0	0	0
	Septal thickening	77 (76%)	24 (55%)	12 (63%)	12 (23%)	5 (5%)
	Bronchial or bronchovascular abnormality	79 (78%)	25 (57%)	10 (53%)	12 (23%)	7 (6%)
	Cavitation	53 (52%)	10 (23%)	2 (11%)	2 (4%)	1 (1%)
	Parenchymal fibrosis	61 (60%)	23 (52%)	11 (58%)	15 (28%)	7 (6%)
	Volume loss	34 (34%)	11 (25%)	5 (26%)	7 (13%)	1 (1%)
	Emphysematous changes	20 (20%)	9 (20%)	3 (16%)	9 (17%)	5 (5%)
	Pleural thickening or fibrosis	20 (20%)	9 (20%)	3 (16%)	9 (17%)	5 (5%)
	Mediastinal or hilar lymphadenopathy	14 (14%)	6 (14%)	3 (16%)	0	0
	Cardiac abnormality	0	0	0	1 (2%)	2 (2%)
Suggestive of current tuberculosis*		83 (82%)	34 (77%)	12 (63%)	5 (9%)	4 (4%)
	Suggestive of current and previous tuberculosis	52/83 (63%)	23/34 (68%)	9/12 (75%)	5/5 (100%)	2/4 (50%)
Suggestive of previous tuberculosis*		59 (58%)	26 (59%)	13 (68%)	13 (25%)	7 (6%)

Data are n (%) or n/N (%).

* Rated by a majority of radiologists as at least somewhat suggestive of current or previous tuberculosis.

## Discussion

In this population-representative cohort of PWTS in a high-burden setting, the 2-year risk of developing tuberculosis was high, even after a baseline evaluation that was interpreted as excluding tuberculosis disease. Approximately a third were diagnosed through additional testing performed at enrolment, and of the remainder, the 2-year cumulative hazard of tuberculosis diagnosis was 0·33. Among PWTS not diagnosed with tuberculosis at baseline, those with atypical initial chest imaging had a particularly high risk of being diagnosed with tuberculosis during follow-up, whereas baseline symptoms showed no prognostic value.

Our report highlights that individuals who receive trace results from tuberculosis screening are at substantial risk of tuberculosis. Although previous studies^3^, ^4^, ^5^, ^7^ showed that 14–43% of screening participants with trace-positive sputum were culture-positive, our results show that culture-negative PWTS are still at significant risk of progressing to tuberculosis diagnosis over time. In this study, 53% of PWTS were diagnosed with tuberculosis within 2 years, and due to incompleteness of follow-up, this proportion is an underestimate of the true positive predictive value of a trace result for having tuberculosis disease within 2 years. Importantly, the positive predictive value could be even higher in populations with a greater underlying prevalence of tuberculosis. For example, although this study used sputum Ultra as an initial test, most screening programmes use Ultra as a confirmatory test among individuals with atypical chest x-ray or symptoms.^3^, ^4^ Since the prevalence of tuberculosis is higher among those who screen positive than among all participants in a screening intervention, the positive predictive value of the Ultra test will also be higher when used to confirm positive screening results rather than as an initial test.

When x-ray is used for screening, our results suggest that the screening step could also increase the probability that people with trace results have or will soon develop tuberculosis, even if further microbiological testing at the time of screening would be negative. Thus, our results support treatment for tuberculosis disease in most PWTS during tuberculosis screening, particularly when trace results are preceded by atypical screening chest x-ray. The appropriate treatment strategy for this form of paucibacillary tuberculosis is uncertain, as low-burden tuberculosis might be cured with shortened regimens.^16^

Our result that many PWTS could be culture negative but are at high risk to progress to advanced disease—while some others could remain without disease over 2 years—can be explained by the understanding of the tuberculosis disease spectrum and natural history. Tuberculosis has a dynamic disease course, and disease burden can fluctuate over time.^8^, ^10^, ^17^ Some culture-positive individuals can have spontaneous improvement and become culture-negative.^9^ This immune-driven reduction in disease burden could leave non-viable *M tuberculosis* that are detectable on molecular tests even after the burden of viable bacteria becomes too small for culture to detect.^18^ However, culture-negative individuals who once had enough mycobacterial burden to produce a positive Ultra result, and who have not received sterilising treatment, could be at high risk of progressing to culture-positive disease. The substantial proportion (nearly half) of PWTS with negative baseline cultures who were nevertheless diagnosed with tuberculosis during this study also suggests that the true specificity of Ultra in community screening contexts, which has already been shown^19^ to be higher than estimates in clinic-based populations, is even higher than previously estimated.

Our results also show the utility of chest imaging in interpreting Ultra trace results. More than half of PWTS without tuberculosis at baseline had chest x-rays interpreted as typical by radiologists, and this subset had a substantially lower risk of developing tuberculosis (cumulative hazard 0·05 at 2 years)—potentially too low to justify empirical treatment for tuberculosis disease, although still higher than the risk in contacts for whom preventive therapy is recommended.^20^ Notably, radiologists interpreting x-ray images in this study had access to paired CT images and imaging results informed clinical decision making. Thus, the estimated HR probably overestimated the ability of x-rays to discriminate early tuberculosis. However, x-ray scores by CAD software still showed utility in identifying individuals diagnosed with tuberculosis among PWTS, particularly among those without previous tuberculosis.

This study has several limitations. Our screening using Ultra might have preferentially selected participants more capable of providing sputum, and PWTS at high risk of tuberculosis might have been more likely to enrol into the study and complete follow-up. We did not induce sputum but accepted suboptimal quality samples if necessary, which might have underestimated microbiological positivity. Differential censoring of participants in the survival analysis could have biased our hazard estimates. In particular, those started on treatment without microbiological confirmation were censored from the analyses that used microbiological positivity as their outcome, but if untreated, they had a high risk of developing microbiological positivity later. Furthermore, if the highest-risk individuals developed tuberculosis early in follow-up, their absence from the cohort during later time periods could have biased our HR estimates. Under our primary definition of tuberculosis, clinical diagnoses might have led to overestimation of diagnosis at baseline, biased the cohort towards low-risk individuals, or overestimated 2-year risk among that cohort. Reassuringly, our estimates were similar under our secondary, microbiological definition, although censoring individuals who started treatment and classifying false-positive microbiological results as positives might have led to underestimation or overestimation of hazards. The small number of events in negative-control participants and PWTS with typical x-rays precluded adjustment for multiple covariates and resulted in wide CIs. Finally, our study was conducted in a single city and screening context, and results could differ in settings with different epidemiology of tuberculosis or different health-care and care-seeking practices.

In summary, we conducted community-based tuberculosis screening using sputum Ultra in Kampala and found that more than half of PWTS were confirmed to have tuberculosis within 2 years. Risk was elevated even among those not initially thought to have tuberculosis after extensive baseline diagnostic procedures. Tuberculosis diagnosis during follow-up was strongly associated with baseline chest imaging abnormalities but not with baseline symptoms. Although further research is needed to identify the most appropriate treatment strategy for these individuals, our findings support tuberculosis treatment for most individuals with trace-positive screening results and atypical chest x-rays. For individuals with negative microbiology and typical chest x-rays, individualised risk–benefit treatment decision making is needed, as their 2-year risk of tuberculosis could still exceed that of contacts who are recommended for tuberculosis preventive treatment.

### Contributors

### Data sharing

The de-identified dataset used for this study and a data dictionary will be available upon reasonable request. Data sharing will be limited to non-commercial research use only. Requests should include a proposal outlining the intended use and method and will be subject to review and approval. Data can be requested through the Vivli repository (https://doi.org/10.25934/PR00011679).

## Declaration of interests

JS, DWD, and EAK report grants paid to their institution to support the work reported in this manuscript. CV reports receiving a trainee abstract travel award from the Infectious Diseases Society of America to attend the 2024 IDWeek conference. All other authors declare no competing interests.
