# Supplementary material for: Long-term risk of tuberculosis among individuals with Xpert Ultra trace screening results in Uganda: a longitudinal follow-up study
Source: Lancet Infect Dis. 2026 Feb;26(2):203–12. doi: 10.1016/S1473-3099(25)00536-5 (PMC12852068; doi:10.1016/S1473-3099(25)00536-5)
Supplement: Supplementary appendix [file mmc1.pdf]

THE LANCET
Child & Adolescent Health

Supplementary appendix

This appendix formed part of the original submission and has been peer reviewed. We post it as supplied by the authors.

Supplement to: Sung J, Nantale M, Nalutaaya A, et al. Long-term risk of tuberculosis among individuals with Xpert Ultra trace screening results in Uganda: a longitudinal follow-up study. *Lancet Child Adolesc Health* 2025; published online Oct 7. https://doi.org/10.1016/S1473-3099(25)00536-5.

Supplementary Appendix Table of Contents

Appendix A. Community-based tuberculosis screening and participant recruitment 3
Appendix B. Selection of negative and positive control participants 4
Appendix C. Sample size calculation for participants with trace-positive sputum 5
Table S1. Minimum two-year cumulative incidence of tuberculosis among participants with trace-positive sputum required to achieve 80% power to detect a difference relative to negative controls... 5
Appendix D. Symptom survey questionnaire at the time of enrollment 7
Appendix E. Symptom survey questionnaire at the time of screening 9
Appendix F. Radiologist interpretation and scoring system for chest imaging 10
Appendix G. Rationale for selecting definitions of tuberculosis 11
Appendix H. Time origins, endpoints, and competing events in survival analysis 12
Appendix I. Safety considerations and procedures for participants enrolled within two years of study completion 13
Table S2. Participants with trace-positive sputum results at screening who had negative microbiological work up but were recommended TB treatment at baseline. 14
Table S3. Participants with trace-positive sputum at screening who subsequently had only negative microbiological results but were recommended TB treatment during follow-up. 16
Figure S1. Cumulative cause-specific hazards of receiving a tuberculosis treatment recommendation (left) or developing a positive microbiological result for tuberculosis (right), among age- and sex-matched pairs of participants with Ultra trace versus negative screening results. 18
Figure S2. Cumulative cause-specific hazards of receiving tuberculosis treatment recommendation (left) or developing a positive microbiological result (right) among individuals with trace screening results, stratified by whether cough was reported at the time of enrollment (top row) or at the time of screening (bottom row). 19
Figure S3. Cumulative cause-specific hazards of receiving tuberculosis treatment recommendation (left) or developing a positive microbiological result (right) among individuals with trace screening results, stratified by baseline chest X-ray results interpreted by computer-aided detection software (qXR v4). 20
Figure S4. Cumulative cause-specific hazards of receiving a tuberculosis treatment recommendation (left) or developing a positive microbiological result for tuberculosis (right), among tuberculosis treatment-naïve participants with trace-positive screening results.. 21
Table S4a. Association between individual characteristics and tuberculosis diagnosis during follow-up, using a treatment-recommendation-based definition of tuberculosis. 22
Table S4b. Association between individual characteristics and tuberculosis diagnosis during follow-up, using a microbiological positivity-based definition of tuberculosis. 22
Figure S5. Distribution of CAD-interpreted baseline chest X-ray scores by initial sputum Ultra results and tuberculosis treatment recommendation, presented as box-and-whisker plots. 23
Figure S6. Receiver operating characteristic (ROC) curves of computer-aided detection software (qXR v4) interpretations of baseline chest X-rays for predicting tuberculosis disease. 24

Table S5a. Sensitivities and specificities of a computer-aided detection software (qXR v4) interpretations of baseline chest X-rays for predicting tuberculosis disease (either at baseline or during follow-up) among individuals with trace-positive screening results. 25

Table S5b. Sensitivities and specificities of a computer-aided detection software (qXR v4) interpretations of baseline chest X-rays for predicting tuberculosis disease (either at baseline or during follow-up) among individuals with trace-positive screening results and no history of prior tuberculosis. 26

Table S6: Number of participants evaluated for enrollment in each arm and their enrollment outcomes 27

Appendix J. TURN-TB Study (Trace Ultra Result iNsight in TB) Protocol 28

Appendix A: Community-based tuberculosis screening and participant recruitment

Screening was initially conducted in partnership with the STOMP-TB (Strategies for Treating, Observing, Managing, and Preventing Tuberculosis) study[1] from February 2021 through August 2021. During this period, screening was primarily door-to-door and took place in three contiguous parishes with an anticipated high burden of tuberculosis. From September 2021 to April 2024, screening was continued by the TURN-TB (Trace Ultra Result iNsight in TB screening) study[2] alone. This screening by TURN-TB was conducted throughout Kampala, in areas with similarly high anticipated TB burden. The TURN-TB study primarily employed event-based screening and selected locations based on TB notification rates from TB registers, as well as data on poverty and crowding.

In addition, participants with trace-positive sputum were also recruited from individuals who participated in similar community-based, symptom-agnostic Ultra testing offered by a national screening program (CAST-TB) in high-risk areas of Kampala.

References:

1. Kendall EA, Kitonsa PJ, Nalutaaya A, Robsky KO, Erisa KC, Mukiibi J, et al. Decline in prevalence of tuberculosis following an intensive case finding campaign and the COVID-19 pandemic in an urban Ugandan community. Thorax. 2024;79(4):325-31.

2. NIH reporter. Who are the Ultra-positive, culture-negative? Understanding the trajectories of individuals in Uganda with trace M. tuberculosis nucleic acid in sputum. Available at: https://reporter.nih.gov/project-details/10466984. Accessed 1 June, 2025

Appendix B. Selection of negative and positive control participants

Negative controls were recruited from among the individuals screened with Ultra by the study, and were selected to be age- and sex-matched to participants with trace-positive sputum. We recruited negative controls of the same sex. For age, we applied a ±2-year caliper for individuals aged 54 or younger, and due to a more limited pool of older individuals, we used progressively broader age windows , increasing the caliper width by ±1 year for every 5-year increment in age beyond 55: ±3 years for ages 55–59, ±4 years for ages 60–64, ±5 years for ages 65–69, and so on. We also controlled the prevalence of HIV in negative control groups. Given that HIV status was not determined until after enrollment, we recruited an additional age- and sex-matched participant with Ultra-negative sputum each time that a negative control matched to an HIV-negative PWTS was found to be HIV positive, thus ensuring that each HIV-negative trace participant had an HIV-negative matched negative control.

The primary analysis is unmatched, as it excludes participants diagnosed with tuberculosis at baseline, resulting in the exclusion of one member of some matched pairs. We then additionally performed a sensitivity analysis, limiting the analysis to pairs in which both participants were not diagnosed at baseline and followed (results presented in Figure S1). For this analysis, we excluded any pair in which either the PWTS or the negative control was diagnosed at baseline or lacked follow-up data. For PWTS participants matched to more than one negative control (due to recruitment of an additional HIV-negative control after the first HIV-positive participant was enrolled), we included the first matched negative control in the survival analysis. If the first matched negative control lacked follow-up data, the second matched control was included instead

Positive controls were also recruited from among individuals screened with Ultra by the study. Consecutive participants with positive Ultra results (greater than trace) were enrolled until 110 participants were reached.

Appendix C. Sample size calculation for participants with trace-positive sputum

We powered our study to detect an elevated risk of incident TB among individuals with trace sputum that was similar to, or greater than, the risk among infected household contacts of an index patient with TB, for whom preventive therapy is recommended.[1] Among the participants in a prior active case finding study in Kampala,[2] the prevalence of Xpert-positive tuberculosis was 2-3 times the national adult average as estimated in a prevalence survey[3] and was higher still when using an event-based door screening approach. We therefore initially anticipated the incidence of tuberculosis among negative controls would be 0.5 to 1% per year, or 2.5 to 5 times the national estimated incidence,[4] translating to a 1 to 2% cumulative risk of TB diagnosis over two years. Based on earlier screening activities,[5] we estimated that 75% of individuals with trace-positive sputum would not have strong evidence of TB at baseline and could be followed for TB during follow-up. We estimated that enrolling 130 participants with trace-positive sputum would result in 80% power to distinguish a two-year cumulative risk of TB of 7-9% (3.5 to 4.5% per year) among individuals with trace-positive sputum compared to negative controls.

We powered our study to detect an elevated risk of incident TB among individuals with trace sputum that was similar or greater than the risk among infected household contacts of an index patient with TB, for whom preventive therapy is recommended.[1] Among the participants in a prior active case finding study in Kampala,[2] the prevalence of Xpert-positive tuberculosis was 2-3 times the national adult average as estimated in a prevalence survey[3] and was higher still when using an event-based door screening approach. Assuming the same relative incidence as relative prevalence, we initially anticipated the incidence of tuberculosis among negative controls would be 0.5 to 1% per year, or 2.5 to 5 times the national estimated incidence,[4] translating to 1 to 2% cumulative over two years. Based on earlier screening activities,[5] we estimated that 75% of individuals with trace-positive sputum would not have strong evidence of TB at baseline and could be followed up for incident TB. We estimated that enrolling 130 participants with trace-positive sputum would result in 80% power to detect a two-year cumulative TB incidence of 7-9% (3.5 to 4.5% per year) among individuals with trace-positive sputum as elevated compared to negative controls.

Table S1: Minimum two-year cumulative incidence of tuberculosis among participants with trace-positive sputum required to achieve 80% power to detect a difference relative to negative controls.

		**PWTS sample size required to reach 80% power**				
		**110**	**120**	**130**	**140**	**150**
**Two-year cumulative incidence of TB among negative controls**	**0.5%**	6.2%	5.8%	5.5%	5.3%	5.0%
	**1.0%**	7.7%	7.3%	**6.9**%	6.6%	6.4%
	**2.0%**	10.1%	9.6%	**9.2%**	8.9%	8.6%
	**3.0%**	12.1%	11.6%	11.2%	10.8%	10.4%
	**4.0%**	13.9%	13.4%	12.9%	12.5%	12.2%

References:
1. World Health Organization. WHO operational handbook on tuberculosis. Module 1: prevention-tuberculosis preventive treatment: World Health Organization; 2024.
2. Kendall EA, Kitonsa PJ, Nalutaaya A, Robsky KO, Erisa KC, Mukiibi J, et al. Decline in prevalence of tuberculosis following an intensive case finding campaign and the COVID-19 pandemic in an urban Ugandan community. Thorax. 2024;79(4):325-31.

3. The Republic of Uganda Ministry of Health. The Uganda National Tuberculosis Prevalence Survey, 2014-2015 Survey Report. 2016.
4. World Health Organization. Global Tuberculosis Report 2024. Geneva, Switzerland: WHO; 2024.
5. Kendall EA, Kitonsa PJ, Nalutaaya A, Erisa KC, Mukiibi J, Nakasolya O, et al. The Spectrum of Tuberculosis Disease in an Urban Ugandan Community and Its Health Facilities. Clin Infect Dis. 2021;72(12):e1035-e43.

Appendix D. Symptom survey questionnaire at the time of enrollment

Questions	***Response options***
Which of the following symptoms do you have currently? (Currently can mean today or within the past few days.)<br>Select all that apply.	Cough<br>Coughing up blood<br>Unexplained fever or chills<br>Unexplained fatigue<br>Drenching sweats at night<br>Shortness of breath<br>Pain in my chest<br>Loss of normal appetite (Anorexia)<br>None of the above<br>Unknown/refused
Within the past twelve months, have you experienced weight loss of more than 5 kg, or enough to make your clothes loose?	No<br>Yes<br>Unknown/refused
Have you noticed any other signs or symptoms of illness that you are concerned about?	
What other symptoms of illness have you noticed?	
Conditional questions for participants who reported any symptoms.	
You said that you currently have a cough. Now looking back in time, for how long have you had this cough? (Interviewer should record answer in weeks.)	
You said that you are currently coughing up blood. Now looking back in time, for how long have you been coughing blood? (Interviewer should record answer in weeks.)	
You said that you currently have a fever or chills. Now looking back in time, for how long have you had fever or chills? (Interviewer should record answer in weeks.)	
You said that you currently have sweats at night. Now looking back in time, for how long have you had sweats at night? (Interviewer should record answer in weeks.)	
You said that you currently have unexplained fatigue. Now looking back in time, for how long have you had unexplained fatigue? (Record answer in weeks, rounding to the nearest week)	
You said that you currently have chest pain and/or shortness of breath. Now looking back in time, for how long have you had chest pain and/or shortness of breath?<br>(Record answer in weeks, rounding to the nearest week. If both symptoms are present, record the number of weeks for the symptom that has been present for the longest duration.)	
You said that you currently have lack of appetite. Now looking back in time, for how long have you had lack of appetite? (Record answer in weeks, rounding to the nearest week)	
How long ago did you first notice your weight loss? (record answer in weeks)	
How do you think your weight is changing now?	I am continuing to lose weight

<table>
<tr><td></td><td>My weight has stabilized
I am regaining or have regained weight
Unknown/Refused to answer</td></tr>
<tr><td>Use the visual below to indicate the severity of your cough in the past week. Please choose the point on this line that indicates the severity of your cough in the past week.
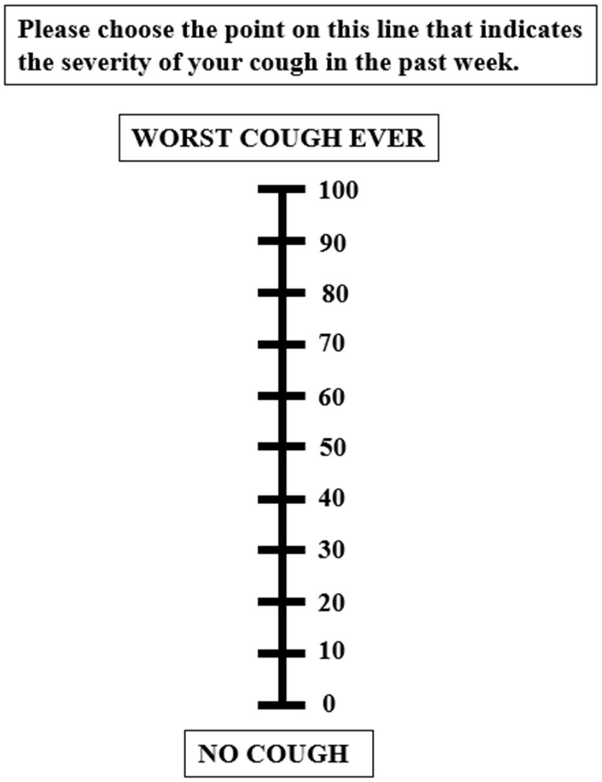
</td><td></td></tr>
</table>

Appendix E. Symptom survey questionnaire at the time of screening

Questions	*Response options*
TB sometimes has no symptoms, so we are testing everyone for TB regardless of whether or not they have symptoms. But we would like to know what symptoms you currently have.<br><br>Do you have cough?	No<br>Yes<br>Unable/unwilling to answer
For how many days have you had cough?	
Do you have sputum?	No<br>Yes<br>Unable/unwilling to answer
For how many days have you had sputum?	
Do you have blood stained sputum?	No<br>Yes<br>Unable/unwilling to answer
For how many days have you had blood-stained sputum?	
Do you have chest pain?	No<br>Yes<br>Unable/unwilling to answer
For how many days have you had chest pain?	
Do you have loss of body weight?	No<br>Yes<br>Unable/unwilling to answer
For how many days have you had loss of body weight?<br>*Approximate from when the participant thinks their weight loss began*	
Do you have fever?	No<br>Yes<br>Unable/unwilling to answer
For how many days have you had fever?	
Do you have excessive night sweats?	No<br>Yes<br>Unable/unwilling to answer
For how many days have you had excessive night sweats?	

Appendix F. Radiologist interpretation and scoring system for chest imaging

Chest X-rays and CT scans from study participants were independently reviewed by two radiologists. If two interpretations were discrepant, a third independent radiologist conducted an additional review. All radiologists were blinded to clinical information and asked to assess the presence of specified abnormalities (e.g., nodules, cavities, fibrosis).

Radiologists were also asked to rate each study's consistency with current and (separately) prior tuberculosis using a four-point scale:

- **3**: Highly suggestive of (current or prior) tuberculosis
- **2**: Somewhat suggestive of (current or prior) tuberculosis
- **1**: Nonspecific finding that could be related to (current or prior) tuberculosis
- **0**: No radiographic evidence of (current or prior) tuberculosis

For each imaging study, we calculated the mean tuberculosis rating across all radiologists. Studies with a mean rating $\geq$1.5 were classified as having imaging findings suggestive of (current or prior) tuberculosis. The 1.5 threshold was chosen to reflect the point at which at least half of the radiologists rated the imaging as at least somewhat suggestive of TB.

Appendix G. Rationale for selecting definitions of tuberculosis

Treatment recommendation was chosen as the primary definition of tuberculosis, as it allowed all evidence of tuberculosis status to be incorporated and was considered more likely to reflect the true disease state — including microbiologically unconfirmed disease — than microbiological positivity alone. Tuberculosis may be bacteriologically-negative (but apparent on imaging and by symptoms), and including expert clinicians' judgment in diagnosing these cases allowed us to avoid microbiological underdiagnosis, given that trace-positive tuberculosis is expected to be paucibacillary.[1] Conversely, a strictly microbiological definition classified six PWTS as having tuberculosis, whose results were not thought to represent true active disease when all available evidence was considered (including treatment history, clinical presentation, and discordance between different microbiological results). Clinicians therefore did not recommend treatment for these individuals, although they were classified as TB under the microbiological definition.

In the primary analysis, where treatment recommendation was used as the definition of tuberculosis, participants who were not recommended for tuberculosis treatment based on evaluations performed at baseline (i.e., upon enrollment) were included in the survival analysis. In the secondary analysis, where tuberculosis was defined exclusively by microbiological positivity, participants without positive microbiological testing results at baseline were included in the survival analysis.

Reference

1. Crowder R, Thangakunam B, Andama A, Christopher DJ, Dalay V, Nwamba W, et al. Diagnostic accuracy of TB screening tests in a prospective multinational cohort: Chest-X-ray with computer-aided detection, Xpert TB host response, and C-reactive protein. Clin Infect Dis. 2024.

Appendix H. Time origins, endpoints, and competing events in survival analysis

The origin and start of the survival analysis were both defined as the time when participants enrolled into the study, which typically occurred within one to two weeks (max 30 days) of screening. The end time was defined as the earliest occurrence of either tuberculosis diagnosis or death from causes other than tuberculosis; for participants who experienced neither event, the end time was the date of the last follow-up visit at which a sputum sample was obtained for microbiological testing.

In the primary analysis, which defined tuberculosis based on treatment recommendation, death from causes other than tuberculosis ("non-TB death") was considered a competing event for survival analysis. Among 77 PWTS who were not recommended for treatment at baseline and were followed for at least one month, no participants experienced a competing event (i.e., non-TB death). Among 108 negative controls who were not recommended treatment and followed for at least one month, one participant (1%) experienced a competing event (death during childbirth).

In the secondary analysis, which defined tuberculosis based on microbiological positivity, both non-TB death and initiation of tuberculosis treatment without microbiological confirmation were considered competing events for survival analysis. Among 78 PWTS without microbiological positivity at baseline and followed for at least one month, no participants experienced non-TB death, but 12 (15%) initiated treatment without microbiological confirmation before completing study follow-up. Among 108 negative controls without microbiological positivity at baseline and followed for at least one month, one participant (1%) experienced non-TB death, and one participant (1%) initiated tuberculosis treatment without microbiological confirmation prior to completing follow-up.

Participants who experienced a competing event were treated as censored in each analysis.

Appendix I. Safety considerations and procedures for participants enrolled within two years of study completion

Three deaths occurred among study participants: One PWTS living with HIV died of tuberculosis at 12 months after missing their six-month study visit and stopping antiretroviral treatment; one negative-control participant died during childbirth; and a positive-control participant died shortly after enrollment, before initiating treatment.

Study progress was monitored semi-annually by an Observational Study Monitoring Board (OSMB). In June 2024, based on preliminary findings, the OSMB recommended that participants with initial trace screening results, no history of prior tuberculosis treatment, and no tuberculosis diagnosis during the study be referred for tuberculosis preventive treatment when they completed study follow-up, and these recommendations were implemented accordingly.

Study follow up ended twelve months after enrolling the last participant. Therefore, not all participants were able to complete a full 24 months of follow-up before the end of the study. All participants had sufficient time to complete 12-month follow-up visits, but 23% (62 out of 267) of participants with initial trace or negative screening results were enrolled less than 24 months before the study ended. For individuals with trace screening results who were still in follow-up at the close of the study, a study visit was conducted in the final month of the study for those enrolled 16-23 months earlier, whereas follow-up time was assumed to conclude at the date of most recent visit for those enrolled ≤15 months earlier.

Table S2. Participants with trace-positive sputum results at screening who had negative microbiological work up but were recommended TB treatment at baseline.

Age, years old	Sex	HIV status	Prior TB	BMI, kg/m^2	TB symptoms at enrollment	Baseline IGRA	Baseline CRP	qXR TB score from baseline CXR*	Baseline CT chest	Rationale for clinical diagnosis at baseline
26	M	Neg	No	17.8	Fever and night sweats for 12 weeks. Weight loss for 4 weeks. No cough.	Not done	<2.5mg/L	0.81	Multiple nodules in upper lobes, and a small cavity seen in the apical segment of the right upper lobe. Multifocal areas of patchy consolidation in the left upper lobe.	Constitutional symptoms, low BMI, CT findings highly consistent with active TB
22	F	Pos	No	22.3	Cough for 2 weeks	Negative	<2.5mg/L	0.03	Unremarkable	Started on treatment by a non-study clinician based on clinical risk (positive HIV and not on ART)
25	F	Neg	No	25.9	None	Positive	<2.5mg/L	0.41	A large, thick-walled cavitary nodule, along with a few additional nodules in the right upper lobe.	Baseline CT highly suggestive of active tuberculosis
40	M	Neg	No	20.3	None	Indeterminate	6.05mg/L	0.67	Bilateral fibrotic and nodular infiltrates.	Repeat sputum Xpert trace-positive and CT concerning for active TB
45	M	Neg	Yes	19.8	Cough for 16 weeks, fever/chills for 8 weeks, weight loss for 28 weeks, shortness of breath and chest pain/shortness of breath for 28 weeks	Positive	7.35mg/L	0.79	Extensive parenchymal fibrosis and bronchiectasis throughout both lungs, multiple nodules and cavities.	Started on treatment by a non-study clinician, based on prior tuberculosis with incomplete treatment. At enrollment the participant reported a history of tuberculosis treatment over a year ago, but he was later found to have not completed the prior course of treatment.
29	M	Neg	No	17.6	Cough, weight loss,	Positive	<2.5mg/L	0.98	Two mass like opacities in the right	Started on treatment by a non-study clinician, after

					and fatigue for 2 weeks; shortness of breath and chest pain for 7 weeks.				lung apex, surrounded by multiple solid nodules.	the participant presented to a health facility with persistent cough and body weakness.
39	M	Pos	No	20.7	Cough, hemoptysis, fatigue, shortness of breath, and chest pain for 2 weeks, and weight loss for 1 week.	Negative	11.49mg/L	0.49	A few random sub-centimeter solid nodules scattered in both lungs	Started on treatment by a non-study clinician, given a new HIV diagnosis.
34	M	Neg	No	19.5	Cough for 3 weeks, fever and chills for 1 week, and weight loss for 4 weeks	Positive	<2.5mg/L	0.99	Multiple centrilobular nodules in both upper lobes, with a small thick-walled cavity is seen in the right upper lobe. Patchy areas of consolidation around the nodules.	Recent TB contact, symptoms, and imaging consistent with active TB.
24	M	Neg	No	19.0	Cough for 4 weeks, weight loss for 15 weeks	Positive	5.01mg/L	0.82	Multiple tiny centrilobular nodules. Patchy consolidation in the apical segment of the right upper lobe.	Recent TB household contact, symptoms, and CT suggestive of early active TB

*qXR TB scores were not available to clinicians making treatment decisions, but they could review chest images.

Table S3. Participants with trace-positive sputum at screening who subsequently had only negative microbiological results but were recommended TB treatment during follow-up.

Age, years old	Sex	HIV status	Prior TB	BMI, kg/m^2	TB symptoms at enrollment	Baseline IGRA	Baseline CRP	qXR TB score from baseline CXR*	Baseline CT chest	Rationale for clinical diagnosis during follow-up.
45	M	Neg	No	21.3	Cough for 2 weeks, fever/chills for 4 weeks, night sweats for 12 weeks, and chest pain/shortness of breath for 12 weeks	Indeter-minate	<2.5mg/L	0.88	Multiple solid nodules in the right upper lobe, including one containing a small cavity. The smaller nodules form branching tree-in-bud opacities.	Treatment was recommended at 3 months, based on a repeat CT which showed progressive peribronchial consolidation with associated centrilobular tree-in-bud nodules in the right upper lobe and increasing ipsilateral hilar lymphadenopathy.
23	F	Neg	No	18.8	Cough and weight loss for 26 weeks, night sweats for 2 weeks, and cough/shortness of breath for 16 weeks	Positive	<2.5mg/L	0.82	A solid lobulated solitary nodule in the left upper lobe associated with ipsilateral hilar lymphadenopathy.	Treatment was recommended at 3 months, based on persistent symptoms, positive baseline IGRA, and a repeat CT scan at 3 months showing a left upper lobe nodule that had decreased in size but remained present.
28	F	Pos	Yes	15.7	Cough and chest pain/shortness of breath for 8 weeks, fever/chills for 4 weeks, night sweats for 8 weeks, and weight loss for 52 weeks.	Negative	11.29mg/L	0.87	Diffuse fibrosis and bronchiectasis in both upper lobes	Started on treatment by a non-study clinician, after discovering that the participant had been diagnosed with Xpert+ TB ~5 months before enrollment and completed only 1 month of treatment.
54	M	Neg	Yes	19.5	Cough for 6 weeks, fever/chills	Positive	11.42mg/L	0.92	Right upper lobe fibrosis. A few random	Started on treatment by a non-study clinician one month after study

					for 3 weeks, night sweats for 6 weeks, weight loss for 2 weeks, and joint pain.				solid nodules in the left lower lobe.	enrollment, due to persistent symptoms and a history of prior incomplete (~4 months) TB treatment four years ago.

*qXR TB scores were not available to clinicians making treatment decisions, but they could review chest images.

Figure S1. Cumulative cause-specific hazards of receiving a tuberculosis treatment recommendation (left) or developing a positive microbiological result for tuberculosis (right), among age- and sex-matched pairs of participants with Ultra trace versus negative screening results. All PWTS and negative controls were matched on enrollment, and this analysis excluded any pair in which either the PWTS or the negative control was diagnosed at baseline or lacked follow-up data. The upper panels (a, b) present the cumulative hazard of tuberculosis diagnosis during follow-up among participants not diagnosed at baseline, estimated using the negative log transformation of Kaplan-Meier survival curves and stratified by participants' initial Ultra results during community-wide tuberculosis screening. The lower panels (c, d) show results only for participants with initial trace-positive Ultra results, stratified by chest X-ray results at enrollment as interpreted by human readers. Shaded areas represent 95% confidence intervals.

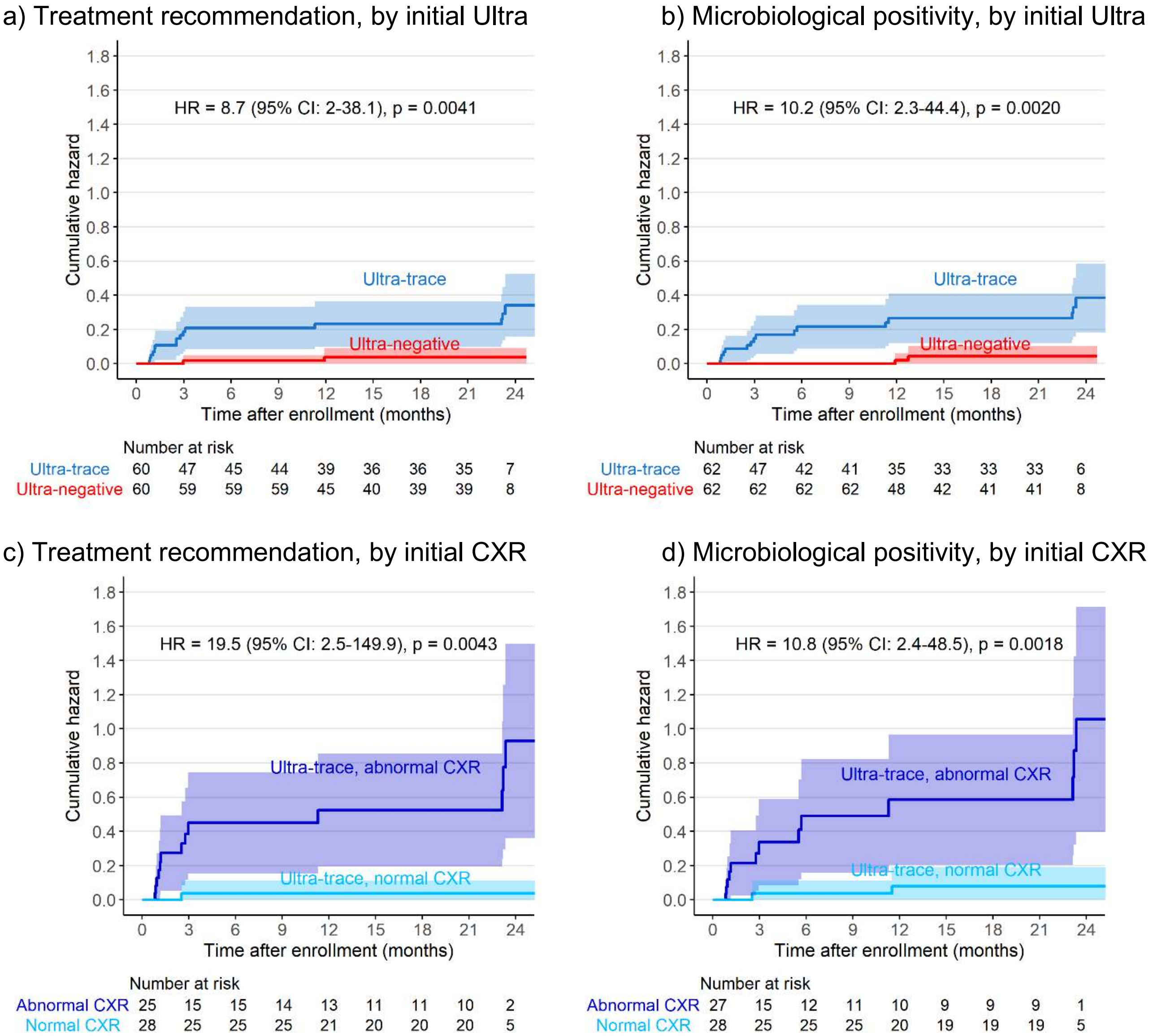

Figure S2. Cumulative cause-specific hazards of receiving tuberculosis treatment recommendation (left) or developing a positive microbiological result (right) among individuals with trace screening results, stratified by whether cough was reported at the time of enrollment (top row) or at the time of screening (bottom row). Abbreviations: HR (hazard ratio); CI (confidence interval); CXR (Chest X-ray)

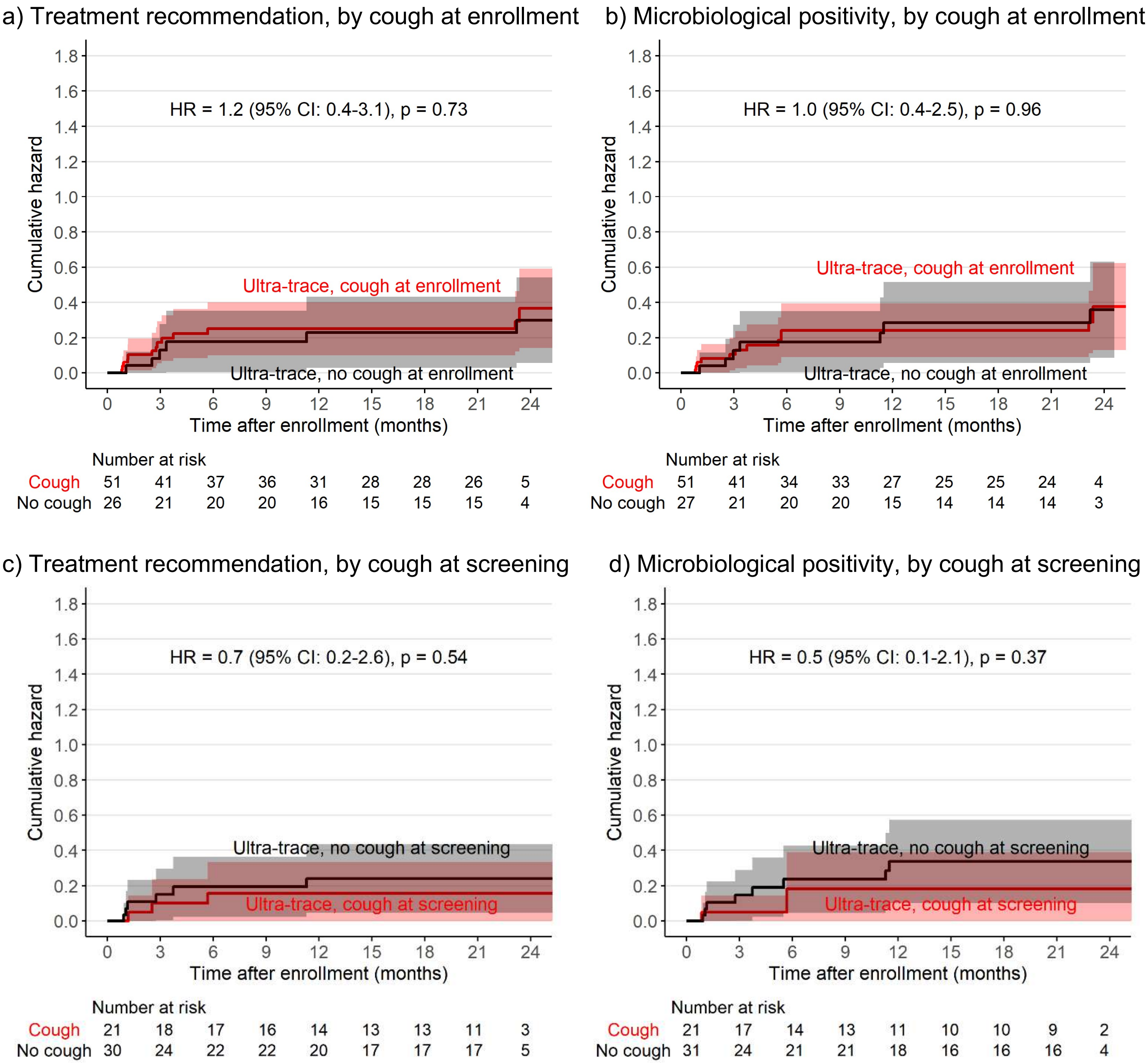

Figure S3. Cumulative cause-specific hazards of receiving tuberculosis treatment recommendation (left) or developing a positive microbiological result (right) among individuals with trace screening results, stratified by baseline chest X-ray results interpreted by computer-aided detection software (qXR v4). Abbreviations: HR (hazard ratio); CI (confidence interval); CXR (Chest X-ray)

a) Treatment recommendation, by CAD threshold of 0.2

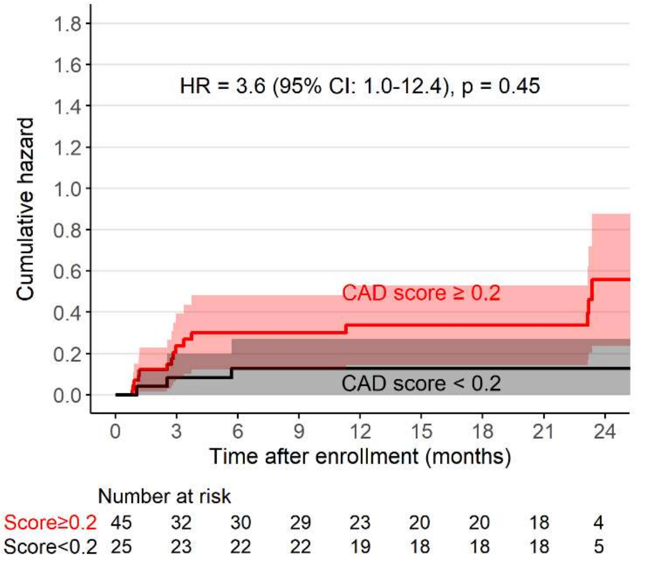

b) Microbiological positivity, by CAD threshold of 0.2

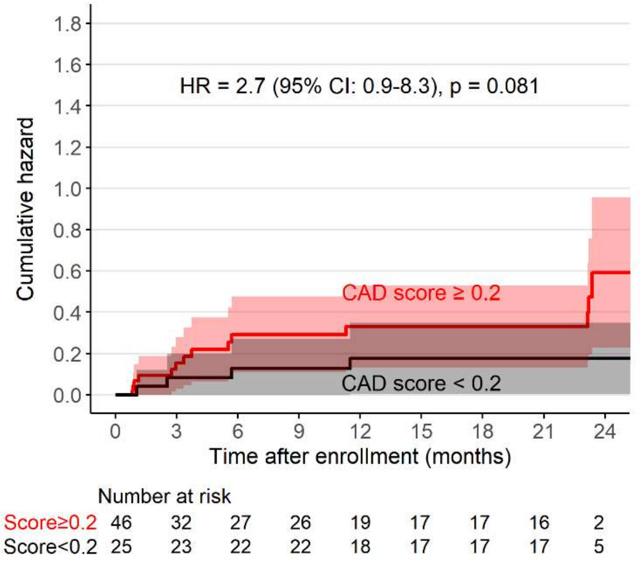

a) Treatment recommendation, by CAD threshold of 0.5

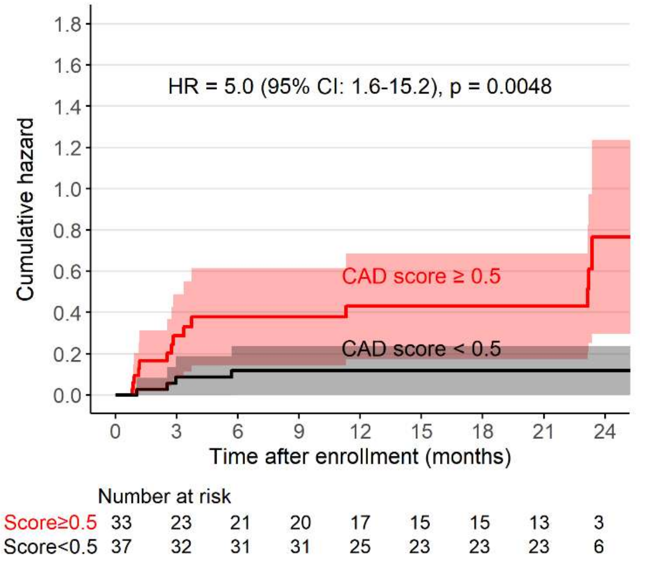

b) Microbiological positivity, by CAD threshold of 0.5

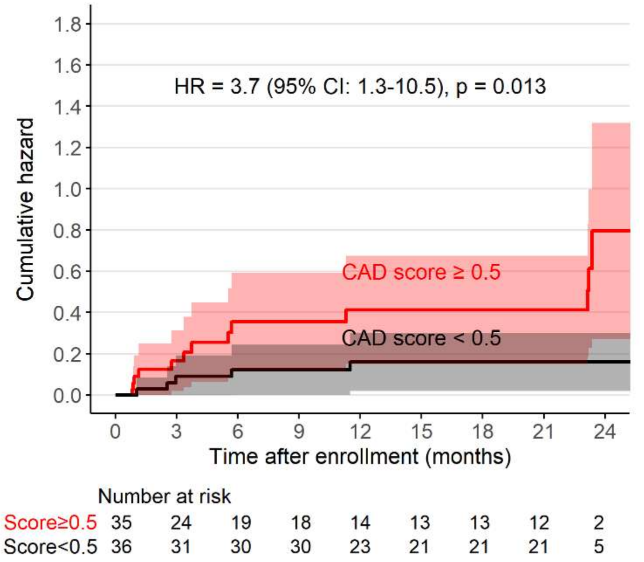

Figure S4. Cumulative cause-specific hazards of receiving a tuberculosis treatment recommendation (left) or developing a positive microbiological result for tuberculosis (right), among tuberculosis treatment-naïve participants with trace-positive screening results. These figures show the cumulative hazard of tuberculosis diagnosis during follow-up among participants with an initial trace-positive Ultra result and no prior history of tuberculosis treatment, stratified by chest X-ray findings at enrollment as interpreted by human readers. Shaded areas represent 95% confidence intervals.

a) Treatment recommendation, by initial CXR

b) Microbiological positivity, by initial CXR

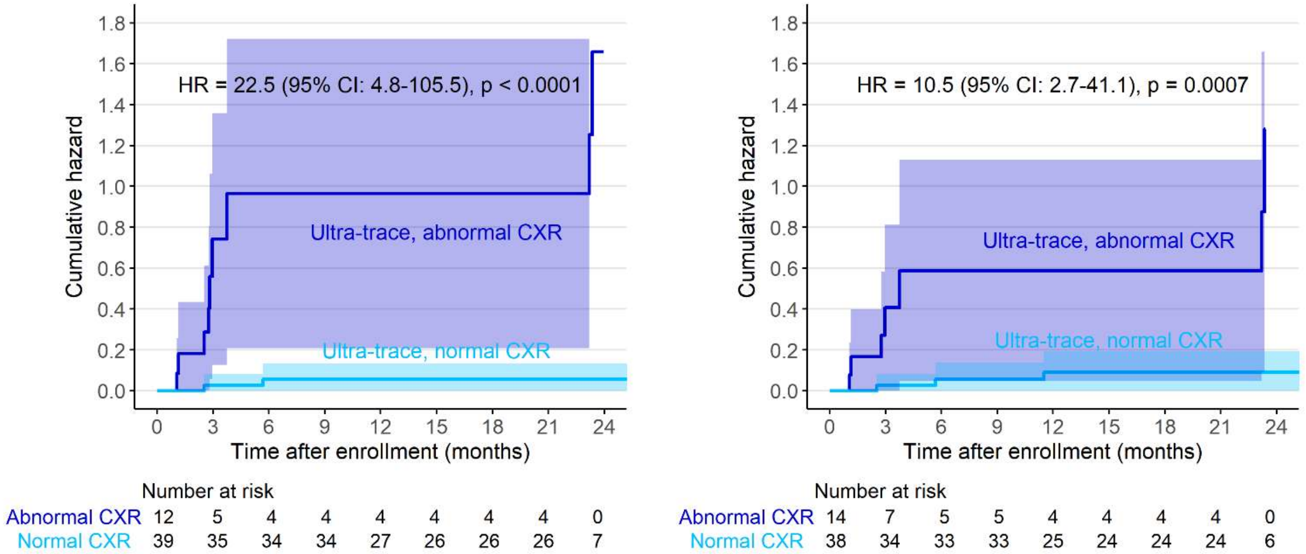

Table S4a. Association between individual characteristics and tuberculosis diagnosis during follow-up, using a treatment-recommendation-based definition of tuberculosis.

	Events in subgroup, total n/N=20/78 (26%)	Hazard Ratio	95% CI	p-value
**Male sex**	12/44 (27%)	1.4	0.6-3.6	0.4775
**HIV infection**	4/10 (40%)	1.9	0.6-5.8	0.2512
**Cough on enrollment**	13/51 (25%)	1.2	0.4-3.1	0.7338
**Any tuberculosis symptom on enrollment**	17/65 (26%)	1.6	0.4-6.8	0.5529
**History of prior TB**	7/19 (37%)	2.1	0.8-5.4	0.1150
**CXR suggestive of active tuberculosis***	5/11 (45%)	2.0	0.7-5.6	0.1867
**CXR with CAD score ≥0.2**	15/45 (33%)	3.6	1.0-12.4	0.0445
**CXR with CAD score ≥0.5**	14/33 (42%)	5.0	1.6-15.2	0.0048
**CXR with any abnormality***	16/30 (53%)	14.6	3.3-63.8	0.0004
**CT suggestive of active tuberculosis***	12/17 (71%)	7.7	3.0-19.5	<0.0001

**Imaging results at baseline evaluation*

Table S4b. Association between individual characteristics and tuberculosis diagnosis during follow-up, using a microbiological positivity-based definition of tuberculosis.

	Events in subgroup, total n/N=20/79 (25%)	Hazard Ratio	95% CI	p-value
**Male sex**	13/45 (29%)	1.9	0.7-5.1	0.1879
**HIV infection**	4/10 (40%)	2.0	0.6-6.0	0.2311
**Cough on enrollment**	12/51 (24%)	1.0	0.4-2.5	0.9644
**Any tuberculosis symptom on enrollment**	17/65 (26%)	1.8	0.4-7.9	0.4268
**History of prior TB**	8/19 (42%)	3.0	1.2-7.5	0.0200
**CXR suggestive of active tuberculosis***	8/13 (62%)	4.8	1.9-12.3	0.0010
**CXR with CAD score ≥0.2**	14/46 (30%)	2.7	0.9-8.3	0.081
**CXR with CAD score ≥0.5**	13/35 (37%)	3.7	1.3-10.5	0.0126
**CXR with any abnormality***	15/32 (47%)	9.6	2.8-33.4	0.0004
**CT suggestive of active tuberculosis***	13/20 (65%)	9.5	3.6-25.2	<0.0001

**Imaging results at baseline evaluation*

Figure S5. Distribution of CAD-interpreted baseline chest X-ray scores by initial sputum Ultra results and tuberculosis treatment recommendation, presented as box-and-whisker plots. Results are shown for (a) all study participants and (b) participants without a prior history of tuberculosis, categorized into five groups: positive controls, PWTS diagnosed at baseline, PWTS diagnosed during follow-up, PWTS not diagnosed with TB, and negative controls. Each box represents the interquartile range (IQR), with the horizontal line indicating the median CAD score. Whiskers extend to the most extreme values within 1.5 times the IQR from the lower and upper quartiles. Values beyond this range are shown as individual dots and represent outliers.

a) All participants

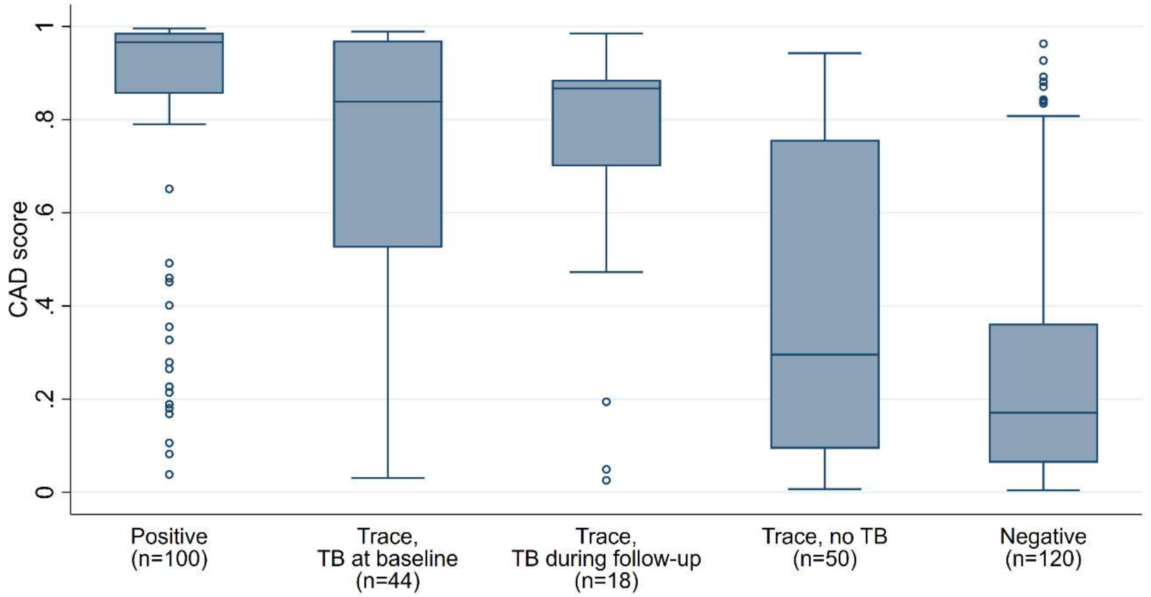

b) Participants without a prior history of tuberculosis

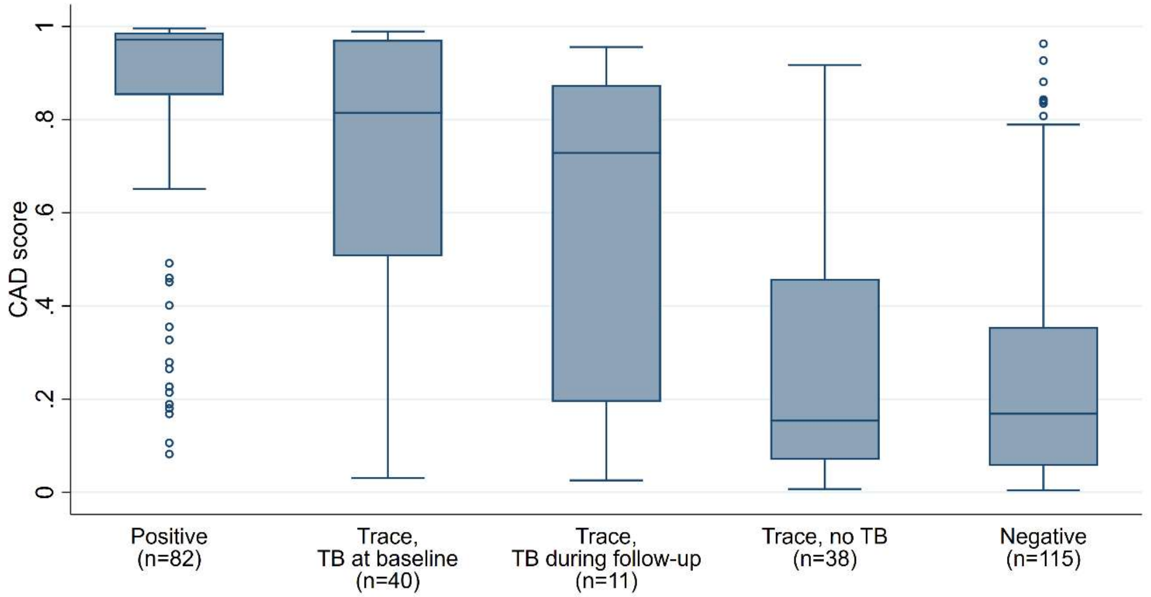

Figure S6. Receiver operating characteristic (ROC) curves of computer-aided detection software (qXR v4) interpretations of baseline chest X-rays for predicting tuberculosis disease. The reference standard for these plots is a treatment recommendation (left panels) or microbiological positivity (right panels), among all individuals with trace-positive screening results (a, b) or only those with no prior history of tuberculosis (c, d). This analysis included participants who completed chest X-rays at enrollment and were either diagnosed with tuberculosis (at baseline or during follow-up) or followed for at least 3 months without a tuberculosis diagnosis. Abbreviations: AUC (area under the curve); CI (confidence interval)

a) Treatment recommendation, all participants

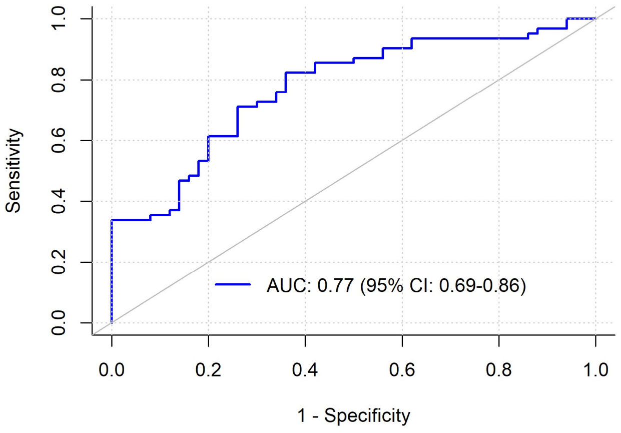

b) Microbiological positivity, all participants

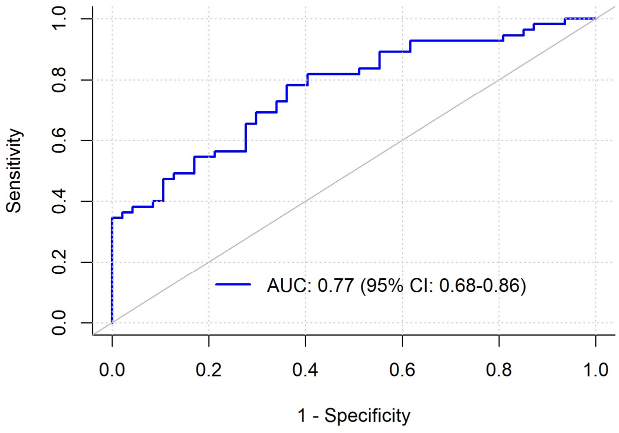

c)Treatment recommendation, no prior tuberculosis

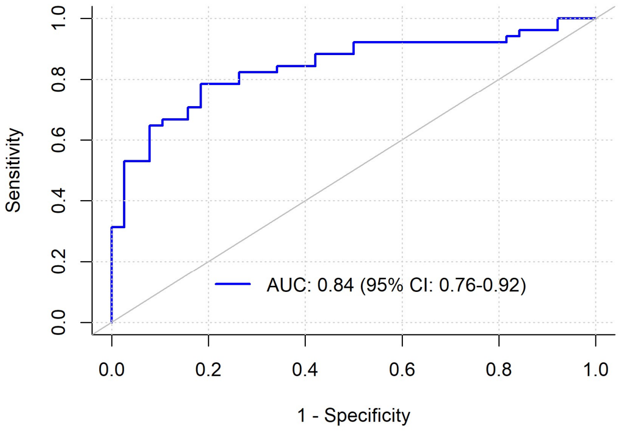

d) Microbiological positivity, no prior tuberculosis

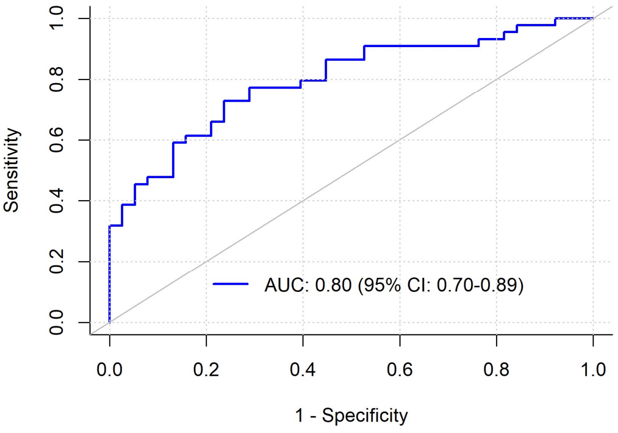

Table S5a. Sensitivities and specificities of a computer-aided detection software (qXR v4) interpretations of baseline chest X-rays for predicting tuberculosis disease (either at baseline or during follow-up) among individuals with trace-positive screening results.

qXR threshold	Treatment recommendation		Microbiological positivity	
	Sensitivity (95% CI)	Specificity (95% CI)	Sensitivity (95% CI)	Specificity (95% CI)
**0.1**	94% (85-97)	26.0% (16-40)	93% (83-97)	26% (15-40)
**0.2**	87% (77-93)	44% (31-58)	85% (74-92)	45% (31-59)
**0.3**	87% (77-93)	50% (37-63)	84% (72-91)	49% (35-63)
**0.4**	85% (75-92)	56% (42-69)	82% (70-90)	55% (41-69)
**0.5**	77% (66-86)	64% (50-76)	75% (62-84)	64% (50-76)
**0.6**	71% (59-81)	72% (58-83)	65% (52-77)	70% (56-81)
**0.7**	66% (54-77)	74% (60-84)	62% (49-73)	72% (58-83)
**0.8**	58% (46-70)	80% (67-89)	55% (41.5-67)	79% (65-88)
**0.9**	35% (25-48)	88% (76-94)	38% (27-51)	91% (80-97)

Table S5b. Sensitivities and specificities of a computer-aided detection software (qXR v4) interpretations of baseline chest X-rays for predicting tuberculosis disease (either at baseline or during follow-up) among individuals with trace-positive screening results and no history of prior tuberculosis.

qXR threshold	Treatment recommendation		Microbiological positivity	
	Sensitivity	Specificity	Sensitivity	Specificity
**0.1**	92% (82-97)	34% (21-50)	91% (79-96)	32% (19-47)
**0.2**	84% (72-92)	58% (42-72)	82% (68-90)	55% (40-70)
**0.3**	84% (72-92)	66% (50-79)	80% (65-89)	61% (45-74)
**0.4**	82% (70-90)	71% (55-83)	77% (63-87)	66% (50-79)
**0.5**	73% (59-83)	82% (67-91)	68% (53-80)	76% (61-87)
**0.6**	65% (51-76)	89% (76-96)	59% (44-72)	84% (70-93)
**0.7**	59% (45-71)	92% (79-97)	55% (40-68)	87% (73-94)
**0.8**	51% (38-64)	97% (87-100)	45% (32-60)	92% (79-97)
**0.9**	31% (20-45)	97% (87-100)	32% (20-47)	97% (87-100)

Table S6. Number of participants evaluated for enrollment in each arm and their enrollment outcomes

Screening Ultra result	Positive	Trace	Negative
Initially selected for enrollment, n	144	144 (125 from a study-conducted screening and 19 from a national screening)	210
Not able to be contacted	8	7	23
Ineligible, n	9<br>• Already on treatment (n=3)<br>• Communication barrier (n=2)<br>• Residence outside the study follow-up area (n=1)<br>• Duplicate screening after enrollment (n=3).	0	4<br>• Communication barrier (n=2)<br>• Residence outside the study follow-up area (n=1)<br>• Not appropriately age-matched to a participant with trace-positive sputum (n=1)
Declined to participate, n	17	9	44
Consenting and enrolled, n	110	128	139

Appendix J. TURN-TB Study (Trace Ultra Result iNsight in TB) Protocol

1. **Abstract**
   a. Provide no more than a one page research abstract briefly stating the problem, the research hypothesis, and the importance of the research.

This project, the TURN-TB study (Trace Ultra Result iNsight in TB), seeks to understand the microbiological origin and clinical significance of "trace-positive" results from a commonly used diagnostic test for tuberculosis (TB), the Xpert® MTB/RIF Ultra ("Ultra") assay. This study initially focused on trace-positive Ultra results obtained during community-based screening for tuberculosis, with subsequent expansion to also evaluate trace-positive Ultra results obtained during TB diagnostic evaluations in outpatient health centers. Although Xpert Ultra is the most widely-used molecular diagnostic test for TB globally, the clinical significance of trace results remains uncertain; in the published literature reviewed in August 2020, only 34% of 91 such patients had other evidence of TB, and WHO guidance notes that "decisions regarding treatment initiation [for patients with Xpert Ultra trace results] should include considerations of the clinical presentation and the patient context" and that evidence to support any particular follow-up testing algorithm is uncertain.[1] Knowing the risk of current and future tuberculosis in such people is expected to aid in providing them appropriate medical care and in understanding the utility of Ultra for finding and treating tuberculosis at its earliest stages. Identifying people with a trace Ultra result but no other signs of active tuberculosis may also provide a unique opportunity to learn about early changes that occur in people who may have incipient TB disease.

Since the start of enrollment in March 2021, the TURN-TB study has recruited participants through community-based TB screening with the Xpert Ultra assay in Kampala. The study was initially conducted in coordination with the STOMP-TB study (JHSPH protocol #IRB00011918 and Makerere School of Public Health IRB #544) and subsequently, independently. Starting in 2022, the TURN-TB study will also recruit participants from health facilities who receive a result of trace on the Xpert Ultra assay. Our enrollment target is a total of 350 individuals with trace-positive Ultra results, including 150 trace-positive participants identified during community-based TB screening.

In Specific Aim 1, we will collect extensive clinical and laboratory data in order to determine what proportion of individuals have evidence of TB disease at the time that they test Ultra-trace-positive, and to understand what other mechanisms might explain the Ultra results of those with no other evidence of active TB. In Specific Aim 2, we will closely follow those without a TB diagnosis, evaluating the incidence of TB and any other TB-related molecular, imaging, and immunological changes that occur over time. In Specific Aim 3, we will use several approaches (decision analytic modeling of individual outcomes, dynamic transmission modeling of population impact, and a public health ethics analysis) to place what we learn from Ultra-trace-positive individuals into context, in order to help public health decision-makers improve how they go about finding people with TB. In an additional analysis focused on trace-positive results in the health facility setting, we will pool data from trace-positive presumptive TB patients with deidentified data from similar trace-positive populations in other settings, for evaluation of predictive models and diagnostic algorithms for classifying which trace-positive patients require treatment for active or incipient TB.

2. **Objectives** (include all primary and secondary objectives)

This project seeks to closely evaluate and follow a cohort of people with Ultra-trace-positive sputum, in order to clarify their current burden and future risk of TB, while gaining insight into the disease dynamics that underlie their Ultra results. Through a combination of community-based screening and recruitment of presumptive TB patients at local health facilities, we propose to identify Ugandan adolescents and adults with Ultra-trace-positive sputum, along with positive and negative controls, for prospective clinical, microbiological, and immunological investigation. We will address the following three specific aims:

Aim 1. Characterize baseline evidence for current or past TB disease and infection among individuals with Ultra-trace-positive sputum. Assessment will include multiple methods to culture or otherwise detect viable *Mtb* bacilli, along with lung imaging, HIV testing, clinical history, symptom survey, cough recording, C-reactive protein measurement, repeat sputum Ultra testing, and contact investigation — with comparisons to age-, sex-, and HIV-matched Ultra-negative controls and to people with positive Ultra and culture results. *We hypothesize that individuals with trace-positive, culture-negative sputum will have minimal TB signs or symptoms, but identifiable subsets will have profiles consistent with resolved prior TB disease and early active TB.*

Aim 2. Longitudinally evaluate the incidence of TB and the dynamics of molecular, imaging, and immunological findings among individuals with trace-positive Ultra but no active TB at study entry. We will (a) conduct two years of follow-up for onset of culture-positive or symptomatic disease; (b) serially monitor IGRA, lung imaging, blood-based inflammatory markers, and quantitative Ultra results for changes associated with new infection, incipient disease, or resolving disease; and (c) identify whole blood transcriptomic and inflammatory cytokine signatures of early or incipient TB. *We hypothesize that TB incidence will be elevated relative to controls and associated with distinctive immunological and imaging characteristics, but most individuals with initially Ultra-trace-positive, culture-negative sputum will remain TB-free.*

Aim 3. Quantitatively and qualitatively explore the implications of trace-positive results and associated patient trajectories for active TB case finding strategies. Using outcome and preference data collected in Aims 1 and 2, we will (a) construct a decision-analytic model to compare the individual-level health implications of possible approaches to TB screening and confirmatory testing, (b) build a transmission model of the comparative population-level impact of TB case finding strategies that accounts for the distribution, dynamics, and detectability of early TB states; and (c) identify criteria that can guide the ethical design of TB case-finding programs. *We hypothesize that Ultra-based screening will have high expected impact but require nuanced implementation, with follow-up preferable to treatment for trace-positive tests meeting certain clinical criteria.*

3. **Background** (briefly describe pre-clinical and clinical data, current experience with procedures, drug or device, and any other relevant information to justify the research)

Significance

Tuberculosis (TB) causes more deaths than any other infectious disease. To reduce the burden of TB globally and in Uganda, there is a need to more consistently and accurately diagnose TB at earlier stages.[2] The Xpert MTB/RIF Ultra nucleic acid amplification test (“Ultra”), now being rolled out

worldwide, can rapidly diagnose TB with high sensitivity. Ultra has potential to play a much-needed role in finding people with undiagnosed TB. Results at Ultra's lowest semi-quantitative result level, "trace," account for much of the assay's enhanced ability to detect TB and may provide an opportunity to characterize TB at earlier stages. However, the usefulness of Ultra is jeopardized by the large number of people (including asymptomatic, treatment-naïve people) whose sputum is positive at the trace level by Ultra, but negative for *M. tuberculosis* by culture.[3] Among symptomatic individuals evaluated in clinical settings with trace Ultra results, more than half have negative results when sputum culture and other extensive investigators are performed,[3,4] yet when it is not possible to routinely perform such investigations or provide close follow up, current WHO guidance recommends treating trace-positive individuals who have no history of recent TB treatment. In a community-based screening context, preliminary data suggest that an even smaller proportion of trace results are accompanied by a positive sputum culture, but it is unclear how best to identify those who do have TB which requires treatment.

The cause of these Ultra-trace-positive but culture-negative results is also uncertain: They might include people who are in the early stages of developing TB disease, people with recent TB that is resolving, people recently exposed to *M. tuberculosis* and possibly infected, or laboratory error. Because the associated risk of developing TB for people with trace-positive Ultra results is unknown, it is unclear how these individuals should be managed. Furthermore, in the context of efforts to achieve earlier diagnosis of TB by screening asymptomatic individuals in the community, it is unclear whether trace-positive Ultra results with negative corresponding cultures represent false-positive tests (and thus should prompt more cautious use of the Ultra assay) or represent very early TB that is likely to progress to advanced TB disease (making people with trace-positive Ultra results the ideal target for efforts at early detection of TB). This study seeks to clarify the current burden and future risk of TB in people with Ultra-trace-positive sputum, while gaining insight into the dynamics that underlie trace-positive Ultra results.

Context and relationship to ongoing studies

The TURN-TB study will be conducted in Kampala, Uganda's capital and largest city. Uganda is designated a high TB/HIV-burden country by WHO and has an estimated urban TB prevalence of 504 per 100,000 adults;[5] 40% of notified TB cases have HIV.[2] Kampala is also home to an extensive diagnostic and research infrastructure including East Africa's only TB Supranational Reference Laboratory, Mulago National Referral Hospital, and Makerere University.

Figure 1: Results of community-based Ultra screening in STOMP-TB

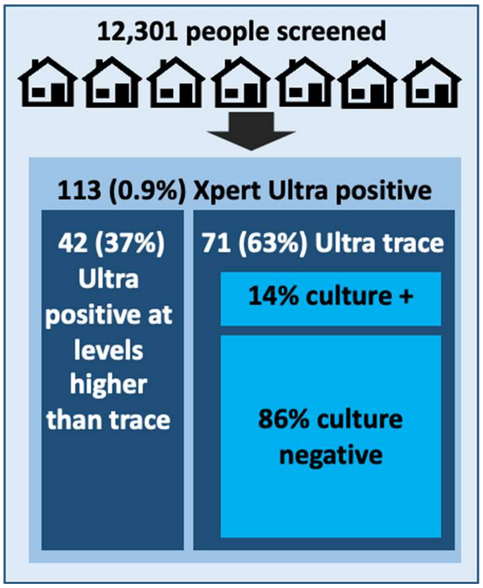

In 2019, our partner study STOMP-TB (Strategies for Treating, Observing, Managing, and Preventing Tuberculosis) conducted intensive community-based case finding in a designated community within Kampala (**Figure 1**); sputum was collected for TB testing from 12,301 individuals, with an interpretable result for 12,032. Of these, 71 (0.60%) were positive by Ultra at the trace level, and 42 (0.35%) were positive at levels greater than trace. Of trace-positive sputa that were cultured, only 14% were *Mtb* culture positive based on a single spot sputum culture. The <u>resulting estimate of the prevalence</u>

of Ultra-positive, culture-negative sputum (500 per 100,000) exceeds both the prevalence of Ultra- and culture-positive sputum in the same community and the national prevalence of TB in Uganda estimated by the 2015 national prevalence survey. Like most prevalent TB in this community setting and other prevalent-TB samples,[6] most trace-positive individuals (89%) tested HIV-negative. It is not yet clear to what extent individuals with this result have early/incipient TB that will require treatment.

4. Study Procedures

a. Study design, including the sequence and timing of study procedures (distinguish research procedures from those that are part of routine care).

The TURN-TB study is structured as a baseline case-control study comparing trace-positive individuals to positive and negative controls, and as a longitudinal cohort study of trace-positive individuals with TB-negative controls. A schematic of study enrollment and follow up is provided in **Figure 2.**

Figure 2. TURN-TB planned enrollment and follow-up schedule

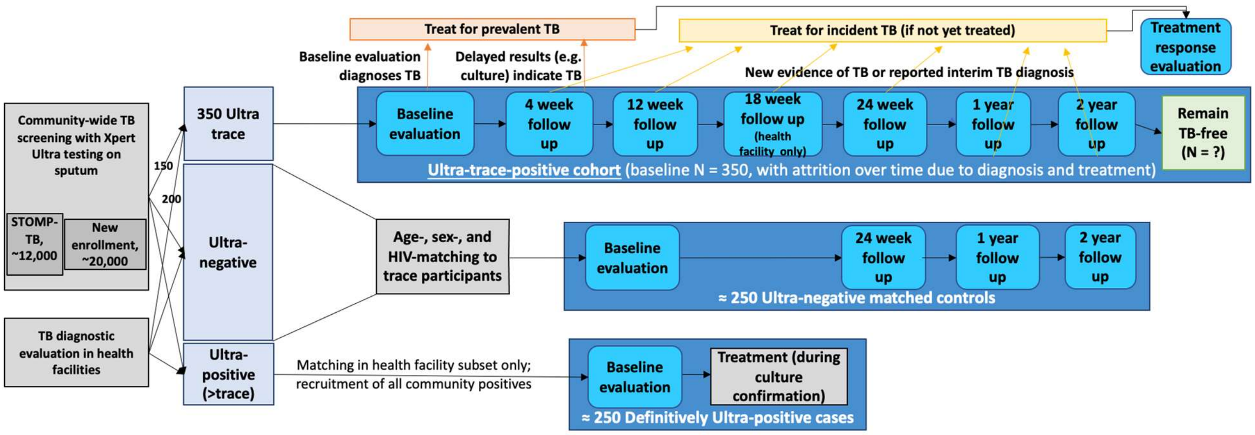

Planned Research Procedures Involving Study Subjects:

Recruitment Process for Community-Based TB Screening

Initial recruitment into community-based TB screening was conducted by the STOMP-TB study. Screening has been done through door-to-door visits in that study's designated study community (the parishes of Kisugu, Wabigalo, and the Namuwongo area of Bukasa parish), as well as through screening events held on a neighborhood level in the same area. After completion of the STOMP-TB study in approximately September 2021, community-based TB screening activities will be conducted by the TURN-TB study team, in additional parishes and zones within Kampala. Recruitment for community-based TB screening will be modeled after a combination of door-to-door and venue-based approaches that was were successfully used by the STOMP-TB study, though will primarily occur through venue-based

approaches. To provide consistency, the two studies share Ugandan and US leadership, and TURN-TB will use approaches developed through STOMP, hire personnel who gained experience with STOMP, and begin work in communities adjacent to the those used in the STOMP-TB study.

All community-based TB screening by the TURN-TB study will take place within the Kampala, Uganda metropolitan area. Initially, screening will be offered in parishes of Makindye division adjacent to the previous STOMP-TB study area, including Kibuli, Kabalagala, and the remaining zones of Bukasa. Later, screening will move into other areas with reasonable proximity to the study's lab and radiology facilities, including parishes of Makindye (both STOMP-TB parishes and others), Kawempe, Rubaga, Nakawa, and Central Divisions, and neighboring zones of Wakiso district.

Within these divisions, zones for screening will be chosen based on estimates of TB burden (priority will be given to zones with higher estimates of TB burden as determined through local public Health Centres or community-level TB risk factors), receptiveness of local leadership to TB case-finding activities, and identification of appropriate venues at which to conduct screening. Study staff will identify promising locations for TB screening within each zone in consultation with local political leaders and community health workers (village health teams, VHTs). Study teams will station themselves temporarily at pre-identified public venues. Selected screening locations within zones may include public transportation hubs, markets, entrances of clinics and pharmacies, community halls, churches and mosques, factories and other large workplaces, and similar public locations where a diverse sample of individuals are accessible. Screening at a given public location will typically be held on a set schedule for several weeks (potentially, with a different location each day of the week, e.g. to correspond to the weekly market schedule). When yield of a given screening location declines and research assistants judge that the location's potential has been saturated, screening activities will be relocated to a new venue. Screening at homes or workplaces using a door-to-door approach (as used by STOMP-TB) may also be offered if community leaders and study staff agree that it is likely to be high yield in a given neighborhood. Word of mouth, local radio announcements, engagement of community leaders, and direct interaction with the public will be used make community members aware that TB screening is being offered and that all are encouraged to participate.

Recruitment will not be based on TB symptoms or individual risk factors; all individuals 15 years and older will be encouraged to participate. Study details will be provided to interested community members via a recruitment script (the first portion of the attached Recruitment and Verbal Consent script), which study staff will read in either English or Luganda.

Community-wide education, which includes a focus on TB stigma reduction, will be provided through information sessions for community leaders and community-based health workers, and community education about TB through visible positioning of study teams in public venues. Study staff will emphasize that all community members (and not only those with specific risk factors) are encouraged to participate in screening.

Individual TB Screening Procedures

All individuals age 15 years and older who are able to provide consent will be eligible for screening. Adolescents ages 15-17 will be asked to assent but will not be enrolled unless a parent, legal guardian, or other legal authority is able to provide consent on their behalf either in person or by telephone. Eligible participants must be capable of providing informed consent or assent and will be required to provide informed consent or assent to participate in the study. We will not attempt to enroll individuals lacking capacity to give informed consent (e.g. intoxicated, emotionally disturbed). Informed consent will be obtained by study staff who have undergone extensive training in the study protocol and human subjects research including patient confidentiality and autonomy.

Due to the brief and low-risk nature of the initial TB screening, the need to enroll a large proportion of the community in order to achieve study validity, the desire to do so with minimal disruption to their daily routines, and the confidentiality risks of asking study staff to carry a large number of consent forms with participant names through their communities, the initial phase of the study will use an oral consent process and will not require witnesses. Consent, if provided, will be documented in the participant's electronic record in REDcap.

Consenting adults and individuals ages 15-17 (whose parents provided informed consent on their behalf) will be asked to provide an expectorated sputum specimen for TB testing. Sputum will be tested for the presence of *M. tuberculosis* DNA using the Xpert® MTB/RIF Ultra ("Ultra") assay; residual sputum from specimens with positive results and from a sample of negative-negative specimens will be used for additional *Mtb* nucleic acid testing. Instruction for expectoration will be provided to participants, including the importance of collecting specimens in a well-ventilated area away from others.

Participation in the screening stage of the study will include collection of contact information (to be stored only temporarily), collection of basic information about age, sex, race, any current TB symptoms and any current TB treatment, and permission to be contacted with results and potentially an invitation to participate in additional research activities if eligible.

Preferred methods of contact for returning negative results (for example, phone call, SMS, email) will be requested from consenting individuals. Individuals with positive test results will be contacted to arrange an in-person meeting during which results will be provided by a research assistant. Contact information will be deleted once Xpert results have been delivered, individuals with positive results have been recruited for further study participation and linked to treatment, and individuals with negative results have been selected and recruited.

Research assistants responsible for screening and recruitment activities will be hired from among the staff who conducted screening activities for the STOMP-TB study. These staff are drawn from the local area and have undergone training in the study protocol, privacy concerns, and communication of sensitive test results.

Case and Control Enrollment in the Community

Results from the initial MTB/RIF Ultra screening are expected within approximately 48 hours after sputum specimens are received by the lab. Results will be used to select individuals for further data

collection. Individuals with trace results will be recruited into TURN-TB's primary trace-positive arm. They will receive an explanation of the uncertain clinical significance of this diagnostic result as part of the consent process. Those with positive results at levels greater than trace will be referred for TB treatment and recruited as positive controls. Negative controls, matched 1:1 based on age and sex to the trace-positive population, will be recruited from among the individuals who tested Ultra-negative. After HIV testing, a small number of additional negative controls may be recruited to ensure that trace-positive cases within HIV have a negative control who is also matched on HIV status (Case-control analyses among HIV-positive individuals will be done in an unmatched manner).

If other health organizations (e.g. the national TB program or nonprofit organizations) conduct community-based TB screening in Kampala using sputum Xpert Ultra as the initial TB test, then individuals among whom their testing detects trace MTB will also be eligible for referral to TURN-TB as trace-positive cases. For these participants, permission to be contacted by a research study will be obtained by either the personnel who conducted the screening or the clinicians at the health center to which these trace-positive participants are referred. The TURN-TB study team will then recruit these trace-positive individuals using the same procedures use for health facility recruitment (as below), but will enroll them as community cases.

At this stage, participants will be asked to provide written informed consent (participant assent and parent/guardian consent, for those aged 15-7), or thumbprints from participants with limited literacy.

Case and Control Enrollment at Local Health Facilities

To ensure the study is able to meet its projected sample size of 350 trace positive participants matched to negative controls, and because of ongoing uncertainty about the optimal management of trace-positive patients in clinical as well as community settings, the study has extended recruitment to individuals who have received a result of trace on a sputum Xpert Ultra diagnostic test in an ambulatory care setting at a local health facility. Health facilities from which trace-positive patients and matched controls may be recruited will include Kisugu (previously the primary enrollment site for STOMP-TB), Kitebi, Kisenyi, Kawaala, Komamboga HCIII, Kiswa HCIV, Naguru hospital, Kibuli hospital, Rubaga hospital, Mengo hospital, Kiruddu national referral hospital, St. Francis hospital, Nsambya, Uganda Cares for Women clinic, and Mulago National referral hospital Wards 5/6.

To provide adequate power for analysis of predictors of prevalent TB and incident TB within the health-facility subgroup of the trace cohort (after pooling with data from similar patients elsewhere; see Statistical Analysis Plan), we will enroll 200 trace participants from Kampala health facilities. Thus, the total number of trace-positive participants enrolled will be 350 (150 from the community and 200 from health facilities). However, the sample size for comparisons to TB-positive and TB-negative controls will remain at most 250 (with negative controls enrolled for all community trace participants and for enough consecutive health facility participants to achieve the target sample size of 250, and with at least 100 positive controls enrolled from each setting). The remaining health facility participants will not be matched to controls, but will undergo the baseline diagnostic evaluation and twelve months of follow-up for incident TB.

Recruitment procedures will be based on those used to recruit health facility-based TB cases for the STOMP-TB study. Study personnel will educate the TB clinical and laboratory staff at selected health facilities on study aims and procedures, and will ask to be notified when any patient (age 15 years or older, and not on treatment at the time of testing) receives a result of trace MTB detected on the Xpert Ultra test. Study personnel will then invite those patients to enroll in TURN-TB.

For trace positive participants recruited from health facilities, study procedures will be the same as for trace-positive participants enrolled through community screening. The managing clinicians will be permitted to initiate TB treatment at their discretion at any time; preliminary discussions suggest that providers will support deferred treatment for many patients if the TURN-TB study provides further diagnostic evaluation and ensures follow-up of abnormal results.

For health facility trace participants who are included in case-control analyses, we will recruit a matched negative control (a patient who received a negative result on the Xpert Ultra and was not empirically treated for TB) and a matched positive control (a patient who received a positive Xpert Ultra result at a level higher than trace), each identified from the Presumptive TB registers at participating health facilities. Because both positive and negative results will outnumber trace results in the health facility setting, both positive and negative controls will be matched on age, sex, and HIV status.

Written informed consent will be obtained as for community cases and controls.

Data Collection
For participants who consent to enroll in TURN-TB as cases or controls, we will perform the following as part of the baseline assessment.

- A **standardized, in-depth interview,** lasting about 45minutes and addressing topics such as medical history, TB symptoms, and TB exposures and risk factors

- Additional questions related to preferences for over- versus under-treatment and the experience of receiving a positive, negative, or indeterminate test result (~10 additional minutes)

- Collection of a spot sputum specimen for
  - Solid and liquid culture and smear microscopy
  - Repeat Xpert Ultra testing

- Collection of one early-morning sputum (or a spot sputum on a separate day, if unable to obtain early morning specimen) for
  - Solid and liquid culture, to be supplemented with *Mtb* culture filtrate[7] to identify differentially-cultivable *Mtb* if liquid culture remains negative after 6 weeks
  - Additional *M. tuberculosis* nucleic acid amplification testing

- Collection of up to 20 ml peripheral venous blood for

- HIV serological testing (SD BIOLINE HIV-1/2 3.0), 1ml, EDTA tube)
- Plasma **CRP** measurement (iChroma, Boditech, run on same specimen as above
- CD4 count measurement (HIV positive participants only)
- QuantiFERON®-TB Gold Plus evaluation of TB antigen reactivity (1 ml x each of 4 tubes)
- Serum for cryopreservation and later use in plasma cytokine/chemokine analysis (10 ml, PET serum tube w/clot activator)
- RNA for cryopreservation and later use in whole blood transcriptome analysis (2.5 ml, PAXgene tube)
- Serum creatinine measurement, for normalization of urine LAM results (trace-positive health facility participants only)

- Collection of urine for
- Point-of-care lipoarabinomannan (LAM) testing for Ultra-trace-positive participants with a positive HIV test result or a previous diagnosis of HIV
- Storage for future evaluation of novel diagnostic assays (e.g. next-generation LAM)

- Collection and storage of a tongue swab for future MTB PCR testing (health facility trace-positive participants only)

- Posteroanterior Chest X-ray to establish a baseline for serial follow-up

- High-resolution non-contrast chest CT for sensitive identification of TB-related abnormalities

- For trace positive individuals, evaluation (history and physical examination) by a medical officer to help guide clinical referral decisions

- Cough recording for approximately 48 hours using the Hyfe Research app on an Android smartphone

For cough recording, participants will be provided with a study smartphone and lavalier microphone at select visits, and asked to keep the phone near them and wear the microphone when possible until they are retrieved by study staff two days later. This app records and processes sounds continuously, and saves a <0.5 second recording whenever there is an "explosive" sound that that exceeds background noise signal by a set threshold. The brief recordings are saved to the phone's hard drive, and uploaded to a secure server managed by Hyfe when a network connection is available. Saved events are analyzed for consistency with cough using a machine learning algorithm, and those above a sensitivity cough probability threshold are reviewed by humans to verify which are true coughs. Recordings are linked to participant records by an ID number documented in participants' REDCap study record. Data associated with each event include the participant ID, a <0.5 second recording, a time stamp, and a GPS coordinate; no other PHI will be stored by or shared with Hyfe. AI analysis and human review of the cough sounds will be done by investigators at Hyfeapp.com, and results returned by ID number to TURN-TB investigators.

At each follow-up visit for trace-positive participants and negative controls, we will repeat a subset of the same evaluations performed at baseline. For negative controls, because the 6-month follow-up time point does not require specimen collection and is primarily for purposes of maintaining contact, the interview will be conducted telephonically unless nonroutine testing (e.g. follow up imaging of a lung nodule) has been recommended by study consultants.

See Table 1 for a full list of study procedures to be performed for individuals in each study arm at each study visit. In addition, for those in the Ultra-trace-positive cohort who are diagnosed with TB, we will repeat certain evaluations at the time of diagnosis (to assess change since last assessment, if >=4 weeks have passed since last evaluation) and at the end of treatment (to assess resolution of symptoms or diagnostic abnormalities that prompted treatment). For participants who initiate treatment for TB, we will review their medical records in the TB treatment and laboratory registers at the relevant health care center to assess linkage to care, treatment completion, and any additional diagnostic testing performed.

Following the baseline evaluation, definitively Ultra-positive TB cases (positive controls) will initiate treatment and will not participate further in the study.

Table 1. Data collection components included at baseline (grey) and in longitudinal (1-24 month) follow-up

	**Study entry†**	**1 mo**	**3 mo**	**6 mo**	**9 mo**	**12 mo**	**24 mo**	**TB diagnosis**	**Post-treatment**
**Clinical/Point of care**									
Interview and detailed symptom survey	X, P, N	X	X	X, N	H	X, N	X, N	X (if not done in past 4 weeks)	X
Subjective preferences and experiences	X, P, N	X		X, N					
Rapid HIV test	X, P, N			X		X, N	X	X (if not tested in past 3 months)	
Urine collection and storage	X	H	H	H					
Urine LAM	X (HIV+)							X (HIV+ and if not done in past 4 weeks)	
Cough frequency recorder	X, P, N		X			X, N		X (if not done in past 4 weeks)	X
History and physical exam by medical officer	X								
Clinical register baseline data abstraction	X, P								
Treatment register outcomes abstraction									X, P
Tongue swab	H								
**Sputum**									

Mycobacterial culture (MGIT and LJ, spot)	X, P, N	H	X	X	H	X, N	X, N	X (if not done in past 4 weeks)	
Mycobacterial culture (MGIT and LJ, AM)	X								
RPF culture, if initial MGIT culture is negative	X								
Repeat Xpert Ultra	X, P, N	X	X	X	H	X, N	X, N	X (if not done in past 4 weeks)	X
Sputum Biobanking	X, P, N		X			X, N		X (if not done in past 4 weeks)	X
**Peripheral Blood**									
CD4 count measurement	X (if HIV+ and no CD4 count documented in the past 1 year or CD4 count pending)								
QuantiFERON TB Gold Plus	X, N, P								
QuantiFERON supernatant storage	X, N								
C reactive protein measurement	X, P, N	X	X	X		X, N		X (if not done in past 4 weeks)	X
PAXGene Biobanking*	X, P, N		X			X, N		X (if not done in past 4 weeks)	X
Plasma Biobanking*	X, P, N		X			X, N		X (if not done in past 4 weeks)	X
Creatinine	H								
**Imaging**									
Chest X-ray	X, P, N		X			X, N	X (if previous imaging was abnormal)	X (if not done in past 4 weeks)	X
High-resolution chest CT	X, P, N **								

X = participants with trace-positive sputum Ultra at study entry; H = participants from health facilities with trace-positive sputum Ultra at study entry; N=negative controls; P=definitively Ultra-positive cases.
†For budgetary reasons, baseline CT and quantiferon testing will be limited to the first ~100 participants in each control arm (a sample size chosen for power to compare baseline characteristics between trace+ participants and controls)
*Cytokine and transcriptomic analyses will be performed on these specimens among a subset of all study groups.
**Abnormal CTs will be repeated at specified intervals if abnormalities cannot be followed adequately by radiograph. Consultants may also request that other procedures (e.g. culture, urine LAM) be performed at additional time points if clinically indicated.

Data collection and storage

Survey and clinical data will be collected using REDCap on an electronic tablet. A data manager will develop and regularly run queries to identify any data discrepancies, which RAs will resolve by consulting the participant or primary source if necessary. Laboratory data and diagnostic results will also be entered into REDCap, with a second RA verifying consistency with the primary lab report/register. Imaging will be digitally stored and interpreted by at least two independent radiologists, using a standardized system and reviewed by a third radiologist when the first two reads are discrepant regarding the probability of active TB.

In addition, the chest X-ray images collected at participants' baseline study visit will be interpreted by the proprietary AI model qXR (QureAI, India). Obtaining qXR scores will allow us to evaluate this commercially available diagnostic product for computer-aided detection of TB in our study population, and will also provide an interpretation of study X-rays that is not biased (as our human radiologists are) by also seeing the corresponding CT images. For this data sharing, DICOM files will be anonymized, assigned random identification numbers that are linkable to study records only by the TURN-TB study team, and shared electronically with QureAI for the purpose of obtaining scoring using their proprietary model. QureAI is providing the analysis as in-kind support for this research, and QureAI will not receive any outcomes data or other clinical data, will not retain the images, will not be allowed to link them to other data sets, and will not participate in publications. As enrollment is ongoing through late 2024, some images may be shared with QureAI for purposes of preliminary analyses once all research support agreements and IRB approvals are finalized, and the remainder will be shared after enrollment has ended. For trace-positive and negative-control subjects, updated and expanded contact information (including names, phone numbers, residential and work addresses or coordinates, and alternate contact numbers such as family members if possible) will be collected at enrollment and updated at each study visit. This will enable the study team to perform ongoing follow up for as long as subjects continue to consent. Personal identifiers will be removed from the database once follow-up is completed. All data will be securely and confidentially stored following the protocols and guidelines specified above.

For purposes of the "Additional analyses of pooled, deidentified data" described in section 7c, including modeling of algorithms for clinical decision-making about diagnostics and treatment after a trace result, a de-identified dataset will be shared with collaborators at the University of Washington for use in collaborative analysis of combined data from Uganda and South Africa.

At the time of publication, the datasets used in analyses will be deposited to a controlled access repository (Vivli, https://vivli.org/about/overview/), along with data dictionaries, enrollment/data collection protocols, and statistical analysis plans. Data will be made available to non-commercial researchers with approved protocols, through a standard process currently being established between Vivli and JHU.

Study timeline

As shown in **Figure 3,** enrollment of individuals for both the Ultra-trace-positive cohort and the positive and negative controls is expected to last approximately 2 years, beginning in the second half of year 1 of

the study. Among those not diagnosed and treated for TB after the initial evaluation, we will conduct 2 years of longitudinal clinical and laboratory follow-up to evaluate clinical outcomes. Whole blood RNA-sequencing and cytokine arrays will be conducted during the last year of participant follow-up, but biospecimens will be collected and stored throughout enrollment and follow-up. Ongoing engagement with the local community and clinicians will occur throughout patient enrollment and follow-up. Analyses will begin in the second half of Year 2 and continue throughout the remainder of the study period, with dissemination of primary results at the end of Year 5.

Figure 3 Study Timeline

	**Year 1**		**Year 2**		**Year 3**		**Year 4**		**Year 5**	
Protocol and Consent forms										
IRB approval										
Data collection instruments										
SOPs										
Kickoff event with community leaders										
Informational meetings with local health facility clinicians										
Ongoing community and clinician engagement										
High-volume TB screening in coordination with STOMP-TB study										
Moderate-paced TB screening (separate from STOMP-TB)										
Enrollment of Ultra-trace-positive individuals										
Enrollment of positive and negative controls										
Two years of follow up evaluations and ongoing contact with participants not yet treated for TB										
Whole blood RNA-Seq and multiple cytokine arrays										
Analysis of baseline prevalence data (Aim 1)										
Analysis of TB incidence and dynamics (Aim 2a-b)										
Analysis of gene expression data (Aim 2c)										
Coding and analysis of individual-level model (Aim 3a)										
Coding and analysis of transmission model (Aim 3b)										
Ethics analysis and generation of action guide (Aim 3c)										
Presentation, publication, and local dissemination of results										

b. If your study involves data/biospecimens from participants enrolled under other research studies with a written consent or under a waiver of consent, please list the IRB application numbers for those studies. Please note: Certificate of Confidentiality (CoC) protections applied to the data in source studies funded by NIH or CDC will extend to this new study if the funding was active in 2016. If this situation applies, Section 36, question 6 in the application will need to be answered "Yes" and "Hopkins Faculty" should be selected in question 7. No other documents are required.

Shared data collection ended in September 2021, when the STOMP-TB study completed enrollment.

c. Study duration and number of study visits required of research participants.

Negative controls will participate in the study for two years. Evaluations will occur at baseline, 6 months, 12 months, and 24 months.

Trace-positive participants will also participate for up to two years. After the baseline evaluation, for those who are not immediately referred to treatment, follow-up evaluations will be scheduled at 4 weeks, 12 weeks, 24 weeks, 1 year, and 2 years. If, at any time during follow up, a participant is diagnosed with TB and initiates treatment, then one follow-up evaluation will be scheduled at the end of treatment. For those who initiate treatment more than one month after the last study visit, a more limited evaluation (including symptom survey and a repeat Ultra) will also be performed at the time of treatment initiation, if possible. Thus, for those who never start treatment, the duration of participation is two years, while for those who are treated, the duration of participation may be less than two years.

Positive controls will complete only a single baseline evaluation at the time of enrollment.

For all types of participants, each study visit will last about an hour and involve an interview, sputum and blood testing, and instruction on the use of a cough monitor (a smartphone attached to a lavalier microphone, which participants will wear for two days following each study visit) and production of an early morning sputum specimen (which study staff will retrieve on the same day that they retrieve the cough monitoring device). If an early morning specimen is not able to be collected, an additional spot sputum will be collected in its place. The participants will also be scheduled to visit a nearby health facility for lung imaging (chest x-ray and/or CT, depending on the visit), possibly on a separate day. We may also contact participants again for study-related purposes (for example, to clarify information that he/she provides to us).

d. Blinding, including justification for blinding or not blinding the trial, if applicable (N/A)

e. Justification of why participants will not receive routine care or will have current therapy stopped.

There is no standard of care for individuals with trace-positive Ultra results in community-based testing. Recommendations do exist for symptomatic, health-care-seeking patient populations: WHO guidelines updated in July 2021 note a low certainty of evidence and recommend that "For patients with Xpert Ultra trace results, decisions regarding treatment initiation should include considerations of the clinical presentation and the patient context (including prior treatment history, probability of relapse and other test results)".[1] When a careful further evaluation is not possible, these guidelines recommend treating those with a trace result and no history of TB treatment – not because all such patients need treatment, but because there is little evidence to support any particular follow-up testing algorithm (such as repeating the Ultra test), and because extensive clinical or diagnostic evaluations are often unavailable. However, in the consent of our

research study, we are able to perform additional evaluations to determine true TB status and provide close follow up to ensure that those who are found to have or develop TB are linked to care.

For a trace Ultra result during community-based screening of a general population, the positive predictive value for culture-positive TB is even lower than in clinical settings. Immediately treating all such people would subject large numbers of otherwise healthy individuals to potentially unnecessary treatment. Additionally, TB treatment is disruptive and costly for patients and carries considerable side effects, and the risk of treatment may outweigh the potential benefits in such patients. Further evaluation (including cultures, clinical and radiographic assessment) to decide which individuals warrant immediate treatment, close follow-up of those who do not, and prompt treatment when clinically indicated, is arguably the ideal management.

Although this extent of evaluation and follow up not feasible on a large scale, it is possible in a research context and is what we will recommend to study participants, although we will not prevent them from seeking treatment sooner if they so choose.

f. Justification for inclusion of a placebo or non-treatment group. (N/A)

g. Definition of treatment failure or participant removal criteria. (N/A)

h. Description of what happens to participants receiving therapy when study ends or if a participant's participation in the study ends prematurely. (N/A)

i. If biological materials are involved, please describe all the experimental procedures and analyses in which they will be used.

We will collect sputum, blood, and in some instances urine and tongue swabs.

Sputum will be used for:

- Solid (LJ) and liquid (MGIT) mycobacterial culture to assess for TB, with MTP64 antigen confirmation of MTB complex species when positive. Liquid cultures that are negative at 6 weeks may be incubated for an additional 2 weeks, either with or without additional of growth-promoting culture filtrate from another *M tuberculosis* culture.
- Smear microscopy
- Xpert MTB/RIF Ultra testing
- TOP PCR testing for the *M. tuberculosis* PonA gene
- Molecular bacterial load assay (*M tuberculosis* 16s rRNA measurement)

Blood will be used for:

- HIV serological testing
- CD4 count measurement in participants with HIV
- plasma CRP measurement
- QuantiFERON®-TB Gold Plus
- Serum creatinine measurement

- For a subset of participants, measurement of multiple cytokine/chemokine expression using bead array
- For a subset of participants, characterization of host transcriptomic signatures using whole blood RNASeq and using a NanoString nCounter platform

Urine will be used for :

- *M. tuberculosis* lipoarabinomannan (LAM) in participants with HIV
- Pregnancy testing prior to x-ray imaging for participants who may be pregnant
- Storage for future evaluation of novel diagnostic assays (e.g. next-generation LAM)

Tongue swab will be used for:

- *M. tuberculosis* molecular diagnostic testing

Mycobacterium tuberculosis culture isolates (obtained through any of the sputum culture methods listed above) may undergo sequencing for inclusion in analyses of TB transmission. Specifically, we plan to sequence approximately 40 culture isolates, collected from participants who live in/near or were diagnosed in/near the study area of the parent STOMP-TB study (JHSPH protocol #IRB00011918 and Makerere School of Public Health IRB #544). These will be used to increase the sample size for analyses of transmission patterns (e.g. extent of genomic clustering, spatial scale of clusters, host characteristics associated with being in a transmission cluster or being identified as a source case in a transmission tree) that were planned as part of the STOMP-TB study.

5. Inclusion/Exclusion Criteria

Study populations will include **(1) an Ultra "trace-positive population", (2) a "definitively Ultra-positive population" (for comparison purposes), and (3) a "negative control population".**

Inclusion criteria for all three groups will include:

- age ≥15 years
- participation in TB testing via the Ultra assay on expectorated sputum, either in community-based screening or in an outpatient TB diagnostic evaluation

In addition, inclusion criteria for each arm will include:

For trace-positive participants:

- a trace-positive sputum Ultra result

For the definitively Ultra-positive population (positive controls)

- a positive sputum Ultra result at a semiquantitative level greater than trace
- At health facilities, matching by age, sex, and HIV status to enrolled trace participants also recruited from health facilities

For the negative control population:

- A negative Ultra result

- At health facilities, matching by age, sex, and HIV status to enrolled trace participants also recruited from health facilities
- In the community (where HIV status is often not known before enrollment), random selection as an age- and sex-matched control of a trace-positive participant, selected as follows: For each enrolled trace-positive participant with a negative HIV result, we will randomly select one age- and sex-matched individual from among those in the same parish with a negative TB screening result. If a consenting negative control tests positive for HIV, he/she will become part of the unmatched negative-control population for trace-positive participants who are living with HIV, and a new age- and sex-matched negative control will be selected and recruited for the HIV-negative, trace-positive participant.

We will exclude participants who:
- are currently incarcerated or institutionalized
- do not speak Luganda or English and do not have an appropriate interpreter available
- are currently taking TB treatment (except for health facility positive controls, who will be permitted to have received up to 7 days of TB treatment at the time of enrollment)

We will include eligible children between the ages of 15 and 17, if both they and their parents/guardian agree to their participation. We have chosen 15 as the age cutoff for screening of asymptomatic individuals because this is the approximate age at which TB incidence begins to rise, at which TB become more likely to manifest as infectious pulmonary disease, and at which patents with active TB are notified to public health authorities as adults. In addition, younger children are less often able to produce sputum, making sputum-based testing (the source of the diagnostic phenotype we are studying) less common, and when younger children do produce sputum of undergo sputum induction, existing diagnostic accuracy studies suggest that false-positive Ultra results (relative to culture) are less common. Therefore, we expect that the same epidemiology and pathophysiology associated with the Ultra-trace-positive sputum will be shared between older adolescents and adults, but may not be relevant to younger children.

6. Drugs/ Substances/ Devices

a. The rationale for choosing the drug and dose or for choosing the device to be used. (N/A)
b. Justification and safety information if FDA approved drugs will be administered for non-FDA approved indications or if doses or routes of administration or participant populations are changed. (N/A)
c. Justification and safety information if non-FDA approved drugs without an IND will be administered. (N/A)

7. Study Statistics

a. Primary outcome variable.

Aim 1 (To characterize baseline evidence for current or past TB disease and infection among individuals with Ultra-trace-positive sputum): **the prevalence of culture-positive tuberculosis among individuals with trace-positive sputum**. This analysis will be stratified by community versus health facility enrollment.

Aim 2 (To longitudinally evaluate the incidence of TB and the dynamics of molecular, imaging, and immunological findings among individuals with trace-positive Ultra but no active TB at study entry): **the relative hazard of microbiologically confirmed TB during follow-up, comparing Ultra-trace-positives who are not diagnosed with TB at baseline to controls with a negative baseline evaluation.** This analysis will also be stratified by community versus health facility enrollment.

An analytic challenge, particularly for our longitudinal analyses, is the classification of participants who start treatment without a definitive diagnosis of TB (for example, who are referred for treatment after a trace-positive result and without negative cultures because a condition such as advanced HIV makes them too high risk, or who choose on their own to seek treatment). These treatment decisions may not represent true TB, but they are also unlikely to be independent of TB risk.
Therefore, for measurements of TB prevalence and incidence, we will consider three definitions of TB:

Culture-positive TB: Participant has a sputum MGIT or LJ culture with growth of *Mycobacterium tuberculosis*. This includes those who may be referred for treatment based on other information while culture results are pending, but whose cultures are ultimately positive.

Microbiologically confirmed TB: Participant has a positive culture as above, OR has a microbiological result other than the trace Xpert (for example a higher positive result on a repeat Xpert Ultra, a positive urine LAM test, or growth of differentially cultivable Mb on culture with resuscitation promoting factor) and is recommended for TB treatment.

Possible TB: Participant is recommended for TB treatment by study physicians or offered treatment by an external physician for any reason, and treatment is not stopped due to an alternative diagnosis or non-response; OR (for longitudinal outcomes) participant dies of TB or of an unknown cause potentially consistent with TB.

b. Secondary outcome variables.

Objective	Outcome	Exposure	Statistical techniques
Identify clinical & epidemiological predictors of prevalent culture-positive TB in trace-positives	Culture-positive TB at baseline (prevalent TB), among trace-positive participants	presence or severity of symptoms, known TB exposures, elevated CRP, HIV status, abnormal lung imaging findings	log-binomial regression
Determine whether differentially cultivable Mtb or detectable RNA are associated with progressive TB	Abnormalities (such as imaging findings, symptoms, elevated CRP, or trends in Ultra cycle threshold while awaiting culture results) that	Positive RPF culture or MBLA, among trace-positive participants with otherwise-negative cultures	Logistic regression

	suggest progressive TB		
Compare clinical TB indicators between trace-positives and controls	baseline symptoms, chest CT abnormalities, CRP levels, cough frequencies	Trace-positives versus positive and negative controls<br>Trace-positives without culture-confirmed TB versus matched negative controls	Fisher's exact tests, Wilcoxon rank-sum tests, log-binomial regression, conditional logistic regression
Understand whether trace-positive participants differ from Ultra-negative controls in prevalence of TB infection or prior TB disease	- IGRA positivity (baseline qualitative and quantitative result, and conversion)<br>- Lung imaging potentially consistent with prior TB per interpreting radiologists	Trace-positives (overall, and limited to those without culture-confirmed TB at baseline) versus matched negative controls	Fisher's exact tests, conditional logistic regression
Describe changes in serial lung imaging among treated and untreated trace-positives	Incidence of meaningful resolution/improvement and of progression, as judged by interpreting radiologists, on:<br>- Any two untreated studies<br>- Baseline versus 12-month study<br>- Pre-treatment versus post-treatment	- All trace-positives, stratified by normal versus abnormal baseline studies<br>- Trace-positives with positive baseline cultures (baseline versus 4-week x-ray)<br>- Trace-positives versus negative controls	Binomial distributions, Fisher exact test, survival analysis
Evaluate transcriptional and cytokine signatures of active and incipient TB population	See below		

c. Statistical plan including sample size justification and interim data analysis.

Our target is for TURN-TB to ultimately enroll 350 participants with Trace-Ultra-positive sputum. Because control matching will be limited to the first 250 trace participants, we anticipate that control matching will result in approximately 270 matched TB-negative controls (see control matching procedure for explanation of >1 control for some cases), and up to 250 positive controls.

Aim 1 (baseline prevalence of culture-positive TB among trace-positive population).

Assessment will be based on sputum specimens collected (for two LJ and two MGIT cultures) as part of the first study visit. Those with fewer than two non-contaminated culture results, or who receive more than one week of TB treatment prior to providing such cultures, will be excluded from the primarily analysis. Uncertainty in prevalence estimates will be based on a binomial distribution. In addition to culture-positive TB, we will also estimate the baseline prevalence of microbiologically confirmed TB and possible TB.

Based on preliminary data, we estimate that approximately 20% of Ultra-trace-positive community participants and 40% of health-facility participants will have positive sputum culture for *Mtb* by one or more culture methods. By enrolling 350 individuals, including 150 from the community setting, we will be able to estimate the TB prevalence among the Ultra-trace-positive population with a 95% confidence interval that has a width of ≤0.1 (i.e., ≤10%) overall and ≤0.18 (i.e., ≤18%) within both community and facility strata.

The case-control sample size of 250 per group will provide high power to detect differences between the Ultra-trace-positive population and controls for secondary outcomes. For example, this sample size will give us 80% power (assuming a two-sided a = 0.05), to detect a mean cough frequency of 0.5 coughs per hour versus 0.4 coughs per hour (assuming previously described variances).[9]

Finally, our increased health-facility sample size of 200 in Uganda will – after pooling with data an additional 200 participants being enrolled under a similar protocol at a South African partner site – will allow us to perform stratified analyses, including precision of ±5-6% in our TB prevalence estimates within key subgroups (HIV+, and HIV-negative treatment-naïve).

Secondarily, we will estimate the prevalence of microbiologically-confirmed TB and of possible TB. Additional secondary analyses are described above.

Aim 2 (incidence of TB, among trace-positive individuals not diagnosed with TB at baseline).

Diagnosis of TB at baseline will include those who are recommended for treatment on the basis of any data collected in the baseline evaluation, or who have a culture specimen collected at the baseline visit that ultimately results as positive. Incidence will be analyzed using a proportional hazards model that accounts for competing risks of mortality and of treatment for TB without microbiological confirmation. We will also estimate the relative hazards of culture-positive TB and of possible TB among trace-positive participants compared to negative controls.

Using data from STOMP-TB and the Ugandan national prevalence survey, we anticipate the incidence of TB in the general population (and thus among negative controls) to be 500 cases per 100,000 person-years. If 150 of our participants come from the community, and 110 of these are not initially treated and enter follow-up successfully, we will have 80% power to detect a TB incidence of 8% overall (4% per year) in the community trace-positive group as an increase compared to negative controls, and to determine that an incidence of 2.5%/year or more is higher than an estimated population average of 0.5%/year. These are similar to the TB risk in child household contacts who are eligible for preventive therapy in our study setting (Martinez et al, Lancet. 2020; 395(10228): 973-984); therefore, if no increase is observed, defining an upper bound to the risk of incident TB will also be a clinically significant outcome. Among the clinical subcohort, if at least 50 trace-positive patients enter follow-up without treatment, comparison to 100 negative controls will allow us to identify a TB incidence difference of 20% cumulative

(10%/year) in the trace group vs 5% cumulative in controls, or of 12% cumulative in the trace group versus 1% cumulative (equal to the background population rate of 0.5%/year) in controls.

For secondary analyses, comparison between prevalent TB (at Trace Ultra or higher levels) and negative controls will provide >90% power to detect a 2-fold difference in gene and cytokine expression associated with early, subclinical TB. If 15% develop TB within two years, we will also have 80% power to identify risk factors associated with 3-fold increased odds of incident TB and to detect 2-fold differences in expression (versus nested controls) of genes or cytokines associated with incipient TB.

Aim 2b) Statistical approach to transcriptome and cytokine expression analyses:
At baseline, we will compare (a) approximately 50 trace-positive, culture-negative individuals and (b) ≥50 individuals with culture-confirmed subclinical TB more broadly (including all community trace-positive, culture-negative individuals and 50 individuals found through active case-finding who are Ultra-positive at higher levels), to (c) at least 50 negative controls.

Longitudinally, specimens from trace-positive individuals who (a) develop incident TB, (b) complete treatment after a baseline TB diagnosis, (c) experience IGRA conversion, and/or (d) complete follow-up without new TB infection or disease, will be compared to (e) specimens from the same individuals at the previous time point and (f) to serial specimens from Ultra-negative controls. The primary longitudinal analysis will depend on the observed outcomes: We will focus on treatment response if the baseline prevalence of TB diagnosis is high, on incident TB (and/or new infection) if the incidence during follow-up is high, and on any difference (or lack thereof) between trace positives and TB-negative controls if a large number of trace-positives complete follow-up with no clinical or microbiological evidence of TB. We will determine whether trace-positive individuals experience greater changes than negative controls, and we will characterize (both in an unbiased manner, and with attention to previously described markers of incipient TB and treatment response) the changes that occur. All analyses will focus on HIV-negative individuals (baseline) or match by HIV status (longitudinally), because of HIV's expected effects on host responses in the few trace-positive participants who are HIV-seropositive.

Differential expression analysis will be performed using DESeq2 to differentiate cases from controls and cases over time. Results will be rlog-transformed for principal component analysis (PCA) to identify demographic and clinical factors associated with differential gene expression. Genes with ≥2-fold differential expression and a Benjamini-Hochberg false discovery rate (FDR) <0.05 will be considered statistically significantly differentially expressed genes (SDEG). We will also separately evaluate the individual differential gene expression and combined predictive accuracy of transcriptomic signatures linked to incipient TB previously[10,11] or prior to our analysis. Hierarchical clustering analysis of SDEG will be performed using Partek Genomics Suite Version 7.0 software (Partek, St. Louis, MO) and further analyzed to determine altered pathway/network using Ingenuity Pathway Analysis (IPA) software (Ingenuity Systems, Redwood City, CA). RT-PCR will be used to confirm key pathways found by RNA-seq to be differentially regulated.

Aim 3 (implications for active TB case-finding strategies). Aim 3 involves modeling, simulation, and ethical analysis rather than human subjects research, so no separate sample size estimate is used.

Additional analyses of pooled, deidentified data

For an additional analysis supported by the Bill and Melinda Gates Foundation, key clinical variables and diagnostic results from the baseline evaluation and first six months of follow-up of health-facility trace-positive patients will be deidentified and pooled with corresponding data from external studies (an analogous prospective cohort of 200 trace-positive patients in South Africa, up to 100 trace-positive individuals incidentally recruited in ongoing multi-site TB diagnostics studies). The combined dataset will be used to:

(a) Identify clinical and diagnostic features associated with microbiologically confirmed TB among Ultra-trace positive patients with and without HIV.

(b) Identify clinical features and diagnostic biomarkers associated with progressive TB disease among ultra-positive patients who did not have definitive evidence of TB at baseline. These include tongue swab PCR and AI interpretation of deidentified digital chest x-ray images, in addition to the diagnostic tests being evaluated for all participants in this protocol.

(c) Use modeling to evaluate context-sensitive algorithms for interpreting trace results, including algorithms that may recommend immediate treatment or specific follow-up tests for trace-positive patients with certain clinical characteristics.

The predictive modeling of this analysis requires a larger sample size than the descriptive aims of the primary project. We estimate that sensitivity or specificity differences of ≥10% could lead to acceptance of a more complex or expensive diagnostic approach (whereas a new algorithm is unlikely to be accepted over the status quo if it does not offer at least this degree of improvement in diagnostic accuracy). Therefore, this analysis is powered to detect sensitivity and specific differences of ≥10% when comparing 2 algorithms. For a 75% correlation between algorithms, achieving this power would require sample sizes of approximately 100 patients classified as having true TB and 100 patients without TB. Because preliminary data suggest that the TB prevalence among the trace-positive clinical population is approximately 40%; therefore, the TB-positive sample is the limiting factor, and enrolling 100 TB-positive patients will require a total enrollment of at least 250 trace-positive patients. If algorithms are to be considered that apply only to certain subsets of patients (e.g. those without HIV), then this sample size would be necessary for that subgroup. Our target enrollment of 200 health-facility trace participants, supplemented by data from external studies, will allow us to reach this target power overall and within key subgroups.

d. Early stopping rules. (N/A)

8. Risks

a. Medical risks, listing all procedures, their major and minor risks and expected frequency.

Our study is designed to pose minimal risk to participants. The potential risks include:

- Breach of confidentiality. Participants will be asked to provide potentially sensitive information (e.g., patient socioeconomic status, TB and HIV status, as well as biological specimens that could be used to extract genetic information), and while every effort will be made to maintain those data

in a confidential and secure fashion, it is possible that such data could become known by individuals other than study personnel.

- Minor inconvenience, particularly from the time required to complete the questionnaire and to travel to the nearest imaging facility.
- Minor discomfort, bruising, or injury from blood draw
- Radiation exposure from chest CT (a single CT, unless abnormalities are found that require CT follow-up) and chest X-rays.

For trace-positive participants, there is also a risk of delayed treatment for TB. In the community setting, the individuals who do not receive immediate treatment will be people who have no definitive TB symptoms or clinical findings and are not seeking medical care; given that we will perform sputum culture and initiate treatment promptly if cultures are positive or signs/symptoms of TB develop, any TB present in these individuals is likely to be treated more promptly as a result of our study than it would be in absence of their study participation. In the health facility setting, patients who would have been treated out of caution may instead be monitored off of treatment due to participation in our study; however, because we will promptly refer any participants with further evidence of TB disease, and because clinicians are likely to choose to treat the highest risk patients regardless of study participation, participation is more likely to result in avoiding unnecessary treatments than in negative consequences of delayed treatment initiation. Trace-positive participants will be actively monitored by the study team and an independent panel of physicians throughout follow-up to identify any signs of TB and refer to clinical care for prompt treatment if needed. See "Trace-Positive Safety and Monitoring" for more information on this independent panel of physicians.

We will not prescribe drugs. Those participants identified as having active TB, and those who qualify for preventive therapy for latent TB or high-risk TB exposure, will be referred to clinical care for treatment, where they will be given drugs with the risk of side effects. However, these drugs will be administered by the clinical staff of TB diagnostic and treatment centers, not our study staff, and the benefits of treatment for TB (including the possibility of averted mortality) far outweigh the risks for those who have active TB. For those with less certain (trace-Ultra-positive) TB diagnoses, our study is designed to determine whether or not they truly have active disease, before exposing them to the potential negative consequences of treatment.

b. Steps taken to minimize the risks.

Data security and participant confidentiality

All study records will be managed in a secure and confidential fashion. Data collected directly from participants will be entered into a REDCap database using password-protected electronic tablets and will be uploaded nightly to a HIPAA-compliant server. Data collectors and managers will be asked to sign a confidentiality agreement prohibiting disclosure of any patient-level information. All communications involving study data will be encrypted.

The local data clerk will review all data collection forms for completion and accuracy, and the data entry process will be audited during all site visits. Error reports from standard queries of the database will be generated and reviewed on a monthly basis, with prompt correction of all errors (including conduct of additional interviews, if necessary).

Laboratory tests will be requisitioned using a study-assigned ID containing no identifiers, which will be linkable to participants by a securely stored key. Laboratory results will be electronically reported to study staff under these study IDs, and then imported into the corresponding REDCap records using an automated script. Imaging will be labeled with these same study IDs for storage and analysis; results (both a standard interpretation, and a structured study-specific enumeration of abnormalities and of consistency with a TB diagnosis) will be imported into REDCap, and images will be stored on a local drive with secure backup. The local drive(s) will be kept in a locked filing cabinet at the central study team offices at Makerere University.

We will also collect human biological specimens, including blood, which contain personal genetic information. The laboratory and bioinformatics personnel who perform RNA sequencing and analyze resulting sequence data as part of this study will not have access to other identifying information such as participant name and address. RNA sequence reads that contain data on individual genetic variation will be shared through a secure platform with only other IRB-approved, HIPAA-compliant studies. Specimens stored in the integrated Biorepository of H3Africa Uganda (IBRH3AU) at Makerere College of Health Sciences will have identifying information removed from their associated metadata, will be accessible only for protocols that have undergone full scientific and ethical review according to IBRH3AU's established protocols (https://ibru.mak.ac.ug), and will be provided with linked de-identified clinical data only to the extent necessary for the proposed research. Blood samples for those participants and time points that are selected for use in transcriptomic and cytokine analyses will be labeled with non-identifying study IDs and shipped to JHU in the final year of the study.

Based on similar principles of data sharing for public good, we will also seek participants' permission to share recorded cough sounds and associated anonymized clinical and laboratory metadata (e.g. previous and current TB treatment status, presence of chest radiographic abnormalities) for inclusion in a secured, international database (operated by Sage Bionetworks) for use in training artificial intelligence algorithms to detect and characterize disease from cough sounds. These brief recordings of <0.5 second explosive sound events cannot be used for participant identification, and all transfer and storage will include only anonymized participant data and will adhere to strict confidentiality requirements and data encryption practices. Only authorized users will have access to the de-identified shared data and Sage Bionetworks will not share participant data with third parties without prior agreement from the principal investigator who oversaw collection of the data.

Datasets for analysis will be de-identified prior to sharing, and identifiers will be destroyed at the end of the study. Potentially identifiable biological sequences (RNA reads) and biological specimens will be stored on a secure server and in a secure biorepository, respectively, and shared only with IRB-approved research studies that include appropriate privacy protections.

Patient safety

For trace-positive participants, the goal is to refer to TB treatment when it is likely to be of benefit, but not to advise treatment when the harms of treatment are likely to outweigh its benefits, relative to an alternative of further monitoring with treatment if new evidence of TB were to develop. This assessment will be made on an ongoing basis. At least every two weeks (and more often if urgent questions arise), the study's advising physicians will review all new study data on trace-positive participants who have not yet initiated TB treatment, as well as confirm completion of previously made recommendations for participants .They will identify any patients who should be referred for clinical evaluation and likely treatment, or who should be recommended for further diagnostic evaluation outside of the study, or who should undergo additional study-related testing (e.g. repeat chest CT). We will also keep any longitudinal healthcare providers of our study participants apprised of their study-related diagnostic results.

For individuals who have a definitively positive Ultra result (*Mtb* detected at greater than trace), or whose further evaluation of a trace-positive result is judged to warrant treatment, our research staff will promptly inform the subject of their result and assist them in identifying a local health facility where the subject would like to seek treatment or further evaluation (which is free to the public in the Ugandan TB program). We will provide the participant (or, with their permission, their healthcare provider) with a clear written explanation of our study, the result(s) that suggested TB, and the results of any other diagnostic testing that was performed. We will also provide contact information so that the managing clinician may discuss with study staff or with the study's physician advisors if they choose. We will do the same for any negative controls with unexpected positive culture results. We will not directly provide or mandate treatment, and therefore we do not anticipate managing adverse events, but we will do all we can to help participants establish linkage to care and to make all relevant study data available and understandable to managing clinicians.

Similarly, for participants who test positive for HIV through our study, or who have a known HIV diagnosis but are not currently in care, we will assist with establishing linkage to care, and we will make relevant study data (including any definitive or possible TB diagnosis, and results of additional diagnostic testing, as well as our plans and schedule for further evaluation) available to HIV treatment providers. Decisions to start TB treatment or preventive therapy will be left to the HIV clinician, with our study staff and advisory panel available to provide more information as needed. For any incidental findings that are judged by study's advising physicians to be of potential medical importance, participants will be advised of the finding and assisted in identifying an appropriate venue for further evaluation or clinical management.

Trace-Positive Monitoring and treatment decisions:
An independent panel of three senior Ugandan physicians (two pulmonologists and a radiologist) will serve as advisors for this study's decisions about whether and when to refer trace-positive individuals to TB treatment. On a biweekly basis, study personnel will compile new patient data for review by the panel. For all trace-positive study participants who have new data since the last review and have not previously been referred to treatment, panelists will be presented with a patient data profile which includes all laboratory data, symptom and cough frequency data, risk factor data such as HIV status, a summary of radiographic findings (with access to images if desired), a list

of any pending studies (e.g. cultures), and the date and content of the next planned data collection time point.

The physician advisory panel will be asked to consider, in light of the data reviewed and the plans for continued close monitoring, whether any participants should be referred immediately for treatment. If the panel recommends that the risks of continued monitoring outweigh the risks of treatment, then the participant will be referred to local TB treatment facilities. The remaining participants — who are not thought to have TB, or for whom the advisory panel agrees there is equipoise regarding the relative risks and benefits of immediate treatment versus continued close monitoring — will continue to be followed closely, with re-evaluations at 1, 3, 6, 12, 24, and 36 months at a minimum. The panel may also make recommendations regarding the need for additional unplanned TB evaluation, such as a follow up chest CT or repeating a contaminated sputum culture. If findings unrelated to TB are identified that require follow up, the study team will make referrals to an appropriate medical provider.

In addition, an Observational Safety Monitoring Board (OSMB) will be convened at the behest of NHLBI. The OSMB will meet every 6 to 12 months to provide oversight of patient enrollment, safety, and data analysis.

Pregnant Women:
Because they are at high risk for TB and will benefit from early TB detection, we will not exclude pregnant women from study participation. However, because of the known association between pregnancy and TB progression, the threshold for treating a person with Ultra trace-positive sputum is expected to be lower in pregnancy. We will inform the study's physician advisory panel of participants' pregnancy status, and we will make TB-related study results available to the obstetric providers of any pregnant participants so that they may choose to treat if indicated. We will not perform chest CT for women known to be pregnant, and we will obtain chest x-rays only if they are expected to be useful for clinical decision-making (e.g. for trace-positive participants without other clear evidence of TB).

c. Plan for reporting unanticipated problems or study deviations.

Research staff will be asked to report any protocol deviations to the study coordinator, who will be responsible for documenting them and reporting them to the PI within two weeks, except in cases where the deviation may have resulted in harm to a participant, in which case thy should be reported to the PI immediately. Deviations will be evaluated based on their potential to harm participants and their potential to compromise the quality or integrity of research findings. Whenever potentially harmful deviations occur, or deviations occur repeatedly, a root cause analysis will be performed, and staff will be retrained or other changes implemented as appropriate.

Research staff will also be required to promptly report any adverse events experienced by participants, and any unanticipated problems (defined as per OHRP guidance as events that are unexpected, possibly related to participation in the research, and increasing the risk of harm to participants), to the study coordinator and/or site PI. In term, the study coordinator and/or site PI will be expected to promptly notify the study PI. Notification of the study PI will be required on

the same day if it is possible that the event constitutes a serious adverse event or an unanticipated (in terms of nature, severity, or frequency) adverse event resulting from study procedures; otherwise, notification will be expected to occur within one week.
The PI will review such events to determine whether they constitute a reportable serious adverse event or unanticipated problem. Such events will reported to the supervising (Hopkins and Makerere) IRBs, to the OSMB, and to NHLBI, according to NHLBI's event reporting timelines https://www.nhlbi.nih.gov/grants-and-training/policies-and-guidelines/nhlbi-adverse-event-and-unanticipated-problem-reporting-policy.

In addition, for all events that are unanticipated and potentially study-related, the PI and study staff will review study procedures and determine whether any modifications should be made for patient safety. Any events that are not otherwise reportable but result in protocol modifications will be reported to the IRB along with the proposed protocol amendment.

d. Legal risks such as the risks that would be associated with breach of confidentiality.

Legal risks of this study similar to those of other studies that collect personally identifying information and health information. Although protections of personal health and other data are generally weaker in Uganda than in the US, Uganda has laws protecting against unlawful disclosure of HIV status (HIV and AIDS Act, 2014) and protecting against general misuse of personal data (Data Protection and Privacy Act, 2019).

e. Financial risks to the participants.

Participants will not be charged for any study activities.

For individuals who have a definitively positive Ultra result (*Mtb* detected at greater than trace), or whose further evaluation of a trace-positive result is judged to warrant treatment, our research staff will promptly inform the subject of their result and assist them in identifying a local health facility where the subject would like to seek treatment or further evaluation. Treatment of TB is free to the public in the Ugandan TB program.

9. Benefits

a. Description of the probable benefits for the participant and for society.

Study participation will be of substantial benefit to participants and their communities. Participation in TB screening will allow people with early TB to be diagnosed and be offered curative treatment before they become ill or die of TB or transmit infection to those around them. Intensive case-finding and treatment of TB at the population level is likely to result in reduced transmission within our study sites, such that the household members and close contacts of participants stand to gain from the study as well.

Detailed evaluation of those with trace-positive results will allow those who require treatment in a timely manner to receive it while avoiding the harms of unnecessary treatment. The knowledge generated about this Xpert Ultra result may also allow others with the same result to be treated appropriately in the future without such extensive follow-up and monitoring.

The community-based study design and associated community engagement activities will be destigmatizing for those found to have TB**.** The study may also allow for earlier HIV diagnosis and treatment in a population at high risk for HIV infection. In a country where 19,000 people (1 in 2,300 residents) die of TB every year,[2] the benefits of early TB detection, while taking measures to avoid overtreatment, outweigh the carefully-managed risks to confidentiality, inconveniences of study participation, and risks of delayed treatment or false reassurance described above.

Importance of the Knowledge to be Gained

The knowledge gained from this study will be of benefit to the larger community, particularly in Uganda, by enabling clinicians and policymakers to make appropriate decisions about TB case-finding and early treatment, and by allowing developers of diagnostic tools to better understand early TB and identify which approaches are likely to detect or prevent future cases. It will also provide detailed information on the progression of TB disease, or lack thereof, in Ultra trace positive individuals, providing new insights into clinical management of these individuals. This increased knowledge has potential to amplify the benefits of the study to the participants' communities and extend a portion of those benefits to similar high-burden communities elsewhere.

10. Payment and Remuneration

a. Detail compensation for participants including possible total compensation, proposed bonus, and any proposed reductions or penalties for not completing the protocol.

For each study visit, participants will be compensated 25,000 Ugandan shillings (approximately $6.70; 4.2 times Uganda's hourly minimum wage) to cover their time and the transportation costs associated with participation. When participants are asked to travel to a separate location for imaging, they will be compensated as for an additional study visit.

There will be no bonuses for study completion or penalties for non-completion.

11. Costs

a. Detail costs of study procedure(s) or drug (s) or substance(s) to participants and identify who will pay for them.

Participants will not be charged for any study activities. For those who require TB treatment, treatment of TB is free to the public in the Ugandan TB program.

References

1 World Health Organization. WHO consolidated guidelines on tuberculosis. Module 3: Diagnosis - Rapid diagnostics for tuberculosis detection 2021 update. Geneva https://www.who.int/publications/i/item/9789240029415.

2 World Health Organization. Global tuberculosis report 2019. Geneva, 2019 https://www.who.int/tb/publications/global_report/en/ (accessed Dec 10, 2019).

3 Dorman SE, Schumacher SG, Alland D, *et al.* Xpert MTB/RIF Ultra for detection of Mycobacterium tuberculosis and rifampicin resistance: a prospective multicentre diagnostic accuracy study. *Lancet Infect Dis* 2018; **18**: 76–84.

4 Mishra H, Reeve BWP, Palmer Z, *et al.* Xpert MTB/RIF Ultra and Xpert MTB/RIF for diagnosis of tuberculosis in an HIV-endemic setting with a high burden of previous tuberculosis: a two-cohort diagnostic accuracy study. *Lancet Respir Med* 2020; **8**: 368–82.

5 The Uganda National Tuberculosis Prevalence Survey, 2014-2015 Survey Report | Ministry of Health. http://health.go.ug/content/uganda-national-tuberculosis-prevalence-survey-2014-2015-survey-report (accessed Feb 1, 2018).

6 Chanda-Kapata P, Kapata N, Klinkenberg E, Grobusch MP, Cobelens F. The prevalence of HIV among adults with pulmonary TB at a population level in Zambia. *BMC Infect Dis* 2017; **17**: 236.

7 Chengalroyen MD, Beukes GM, Gordhan BG, *et al.* Detection and Quantification of Differentially Culturable Tubercle Bacteria in Sputum from Patients with Tuberculosis. *Am J Respir Crit Care Med* 2016; **194**: 1532–40.

8 Honeyborne I, McHugh TD, Phillips PPJ, *et al.* Molecular Bacterial Load Assay, a Culture-Free Biomarker for Rapid and Accurate Quantification of Sputum Mycobacterium tuberculosis Bacillary Load during Treatment. *Journal of Clinical Microbiology* 2011; **49**: 3905–11.

9 Proaño A, Bravard MA, López JW, *et al.* Dynamics of Cough Frequency in Adults Undergoing Treatment for Pulmonary Tuberculosis. *Clin Infect Dis* 2017; **64**: 1174–81.

10 Schumacher SG, Denkinger CM. Diagnostic Test for Incipient Tuberculosis: A Step Forward, Many More to Go. *Am J Respir Crit Care Med* 2018; **197**: 1106–7.

11 Scriba TJ, Penn-Nicholson A, Shankar S, *et al.* Sequential inflammatory processes define human progression from M. tuberculosis infection to tuberculosis disease. *PLoS Pathog* 2017; **13**: e1006687.
